# A Reaction‐Diffusion Frame for Accessing Metabolic O_2_ Fluxes in Single Microalgal Cells with Low‐Cost Wide‐Field Imaging of Nanosensor Luminescence Lifetime

**DOI:** 10.1002/advs.202510903

**Published:** 2025-08-29

**Authors:** Hélène Merceron, Eliora Israelievitch, Valentine Rollot, Théo Villarubias, Xiaojiang Xie, Thomas Le Saux, Karim Benzerara, François Guyot, Alix Boulouis, Emmanuelle Marie‐Bègue, Laurent Thouin, Ludovic Jullien

**Affiliations:** ^1^ CPCV Département de chimie École normale supérieure PSL University Sorbonne Université CNRS 24 rue Lhomond Paris 75005 France; ^2^ Muséum National d'Histoire Naturelle Sorbonne Université UMR CNRS 7590. Institut de Minéralogie de Physique des Matériaux et de Cosmochimie (IMPMC) 4 Place Jussieu Paris 75005 France; ^3^ Chloroplast Biology and Light Perception in Microalgae UMR7141 CNRS Sorbonne Université Paris France; ^4^ Department of Chemistry The Hong‐Kong University of Science and Technology Clear Water Bay Kowloon Hong Kong China

**Keywords:** chlamydomonas reinhardtii, lifetime imaging, oxygen nanosensor, photosynthesis, reaction‐diffusion

## Abstract

In cells, many small molecules are membrane‐permeant. This feature opens a road to analyze their flux of production or consumption by quantitatively interpreting the map of their extracellular concentration within a reaction‐diffusion frame. Here, this approach is implemented with a new wide‐field lifetime imaging protocol applied to single microalgae cells sparsely deposited on an agarose pad loaded with a luminescent dioxygen (O_2_) nanosensor. The resulting maps are processed to access the spatial distribution of the O_2_ concentration in the plane of the cells. After fitting the data, the cellular O_2_ flux is extracted, evidencing a span of magnitudes and angular dependencies in the balance between photosynthesis and respiration. Beyond pointing to the disparity of individual behavior within the same colony, this work validates a simple approach for characterizing metabolic fluxes of membrane‐permeant molecules down to the single‐cell level.

## Graphical abstract

Individual *Chlamydomonas reinhardtii (C. reinhardtii)* living cells on an agarose pad containing luminescent O_2_ nanosensors are imaged with a simple wide‐field protocol of lifetime imaging. The resulting maps of phosphorescence lifetime are analyzed within a reaction‐diffusion frame to access the distribution of metabolic O_2_ fluxes generated by single microalga cells.

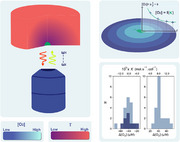

## 1 Introduction

Small membrane‐permeant molecules such as gases (e.g., O_2_, CO_2_, NO, H_2_S, H_2_, CH_4_, and C_2_H_4_) are involved in multiple cellular metabolisms, where they ensure key functions such as electron initial donor or final acceptor, carbon source, or signaling molecule.^[^ ^1^ ^]^ Hence, these gases can fruitfully be used to report on the physiological state of cells.^[^ ^2^ ^]^ They can also intrinsically represent a target of economic interest, for the production of bio‐hydrogen by microorganisms for instance.^[^ ^3^, ^4^ ^]^ Most current investigations of their metabolic fluxes have been led at the population level, involving large numbers of cells, thereby masking the performance of individuals that could exhibit singular and attractive behaviors.

Single‐cell physiology has recently attracted attention by delivering individual metabolic profiles, which enable the measurement of heterogeneity within a population.^[^ ^5^, ^6^, ^7^, ^8^ ^]^ In this emerging field, most experimental approaches are invasive (e.g., omics, mass spectrometry) or demand organism modifications (e.g., by specific labeling with fluorescent proteins). By contrast, in this work we designed and evaluated, in single living microorganism cells, a non‐invasive method that exploits the essentially free permeation of a small and uncharged gas molecule across cell membranes to unravel its metabolism.

**Figure 1:**
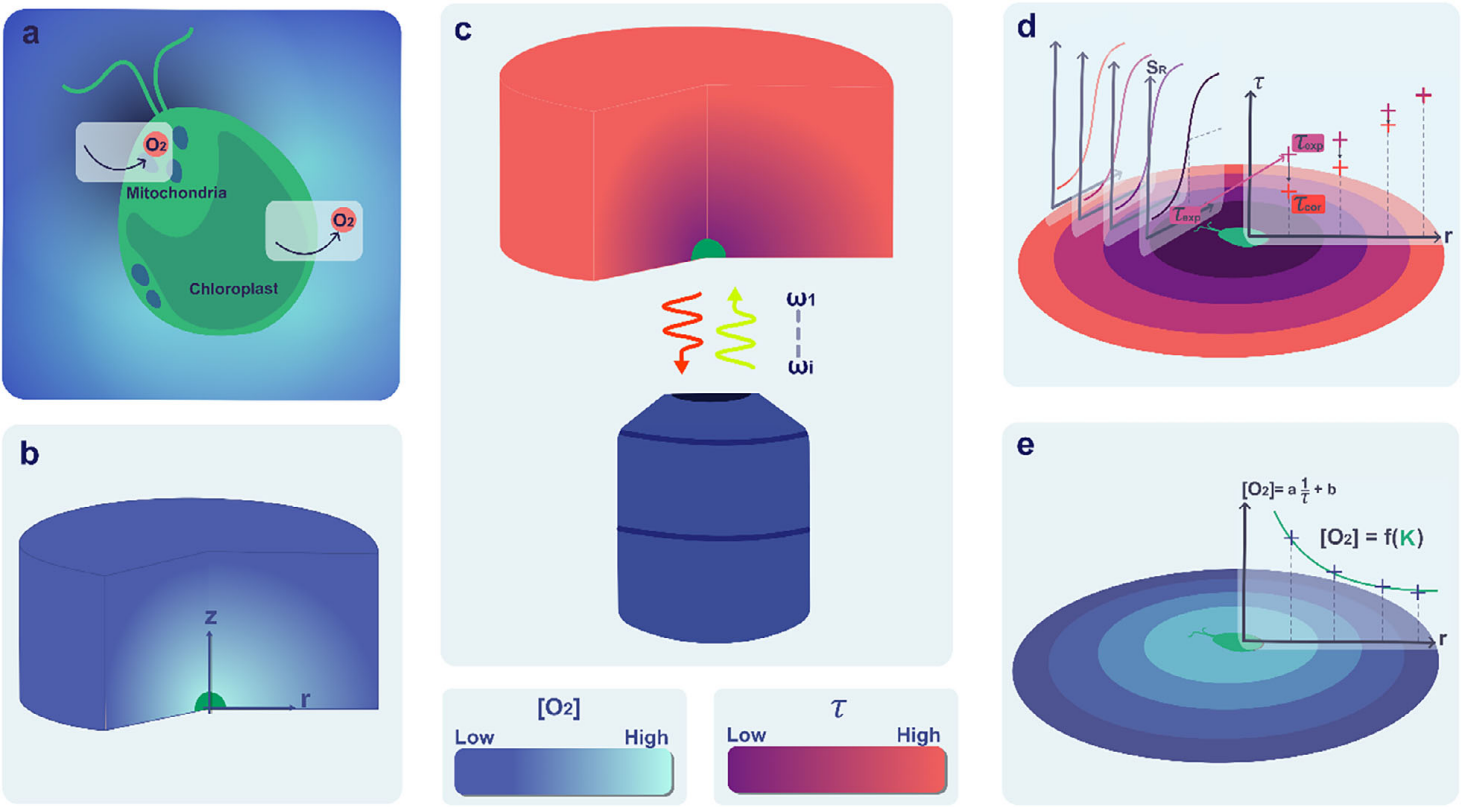
Steps toward the metabolic O_2_ flux in a single microalgal cell from wide‐field imaging of the luminescence lifetime of an O_2_ nanosensor. a) In a microalgal cell (here *Chlamydomonas reinhardtii (C. reinhardtii)*, photosynthesis and respiration that take place in the chloroplast and mitochondria, respectively, generate a 3D spatial profile of membrane‐permeant molecules such as O_2_; b) Once immobilized on an agarose pad loaded with the nanosensor, a single microalgal cell drives a stationary 3D profile of O_2_ concentration; c) The O_2_ concentration‐dependent luminescence lifetime of the nanosensor is interrogated in microscopy with the RIOM protocol; d) In epifluorescence microscopy, it results in an experimental map of luminescence lifetime, which is a 2D projection of the 3D profile of luminescence lifetime over the thickness of the agarose pad. Assuming isotropic O_2_ diffusion, the map of luminescence lifetime of the nanosensor located in the cell plane is retrieved after data processing; e) The latter map eventually yields the map of O_2_ concentration in the cell plane from which the O_2_ cellular flux is extracted by fitting.

Photosynthesis and respiration are two major sets of processes exploited by living beings to fuel their metabolism. In photosynthetic organisms, these processes take place in chloroplasts and mitochondria, respectively (**Figure** **1**a). These organelles exchange small molecules^[^ ^9^, ^10^ ^]^ such as O_2_, which is involved in multiple regulatory pathways.^[^ ^11^ ^]^ Several probes and protocols currently exist for O_2_ sensing,^[^ ^12^, ^13^, ^14^ ^]^ which have been implemented in the context of photosynthetic organisms.^[^ ^15^, ^16^, ^17^, ^18^, ^19^ ^]^ Among these, electrochemical detection and optical sensors have been the most commonly exploited. In the first approach, the sensing cathode reduces O_2_ to hydroxide ions, generating a current linearly proportional to the O_2_ concentration.^[^ ^20^ ^]^ In optical sensing, the decrease in the intensity and lifetime of luminescence of an O_2_‐sensitive probe yields information about the O_2_ concentration.^[^ ^21^ ^]^

In photosynthetic organisms, O_2_ concentration has mostly been measured locally with needle‐type microelectrodes or microoptodes to report on dynamic aspects of photosynthesis.^[^ ^22^, ^23^, ^24^ ^]^ A dioxygen‐selective self‐referencing electrochemical microelectrode has been exploited to quantitatively measure the O_2_ concentration profile around a single green algal cell.^[^ ^25^ ^]^ An absolute luminescence lifetime‐mediated measurement of O_2_ concentration in O_2_‐sensitive phosphorescent microbeads‐injected cells of green plants has been reported.^[^ ^26^ ^]^ 2D O_2_ distribution has also been imaged within a photosynthetic sample. Electrochemical imaging of the dynamics of light‐induced O_2_ evolution has been reported in leaves with large‐scale integration‐based amperometric sensors^[^ ^27^ ^]^ and down to the single‐cell level with scanning electrochemical microscopy.^[^ ^28^, ^29^, ^30^ ^]^

Alternatively, 2D maps of O_2_ concentration have also been obtained either by adopting an emission ratiometric sensing scheme involving an O_2_‐sensitive metal complex and a reference fluorophore embedded in a nanosensor^[^ ^31^, ^32^ ^]^ or a planar film,^[^ ^33^ ^]^ or by luminescence lifetime imaging of an O_2_‐sensitive metal complex contained in a polymeric film.^[^ ^34^, ^35^ ^]^ In the present study, we demonstrate that the spatial distribution of O_2_ concentration around microalgae cells can be quantitatively imaged down to the single‐cell level to retrieve physiological information with a simpler setup.

We recently reported about the Rectified Imaging under Optical Modulation (RIOM) protocol, which enables wide‐field imaging of the phosphorescence lifetime of metal complex‐loaded microbeads using a regular epifluorescence microscope equipped with a standard camera.^[^ ^36^ ^]^ Here, we implement RIOM to image the phosphorescence lifetime of an O_2_‐sensitive nanosensor contained in an agarose pad on which microalgae cells have been sparsely deposited. The resulting information is processed in a reaction‐diffusion frame to access the spatial distribution of O_2_ concentration around single cells and extract the cellular O_2_ flux. We evidence that the cells exhibit a range of levels and angular dependences of the photosynthesis‐respiration balance, which points to the disparity of individual behaviors within a single clonal population. This work additionally brings validation of a simple method that is further discussed to characterize metabolic fluxes of permeant molecules down to the single‐cell level.

## 2 Results

### 2.1 The Concept

At steady state, the outflux or influx of O_2_ driven by photosynthesis in the chloroplast and respiration in mitochondria generates a stationary 3D O_2_ concentration pattern around a microalgal cell (Figure 1a,b).^[^ ^37^ ^]^ In our experiments, the cell is deposited at the bottom surface of a thick agarose pad homogeneously loaded with a phosphorescent O_2_ nanosensor. The luminescence response of the nanosensor induced by the RIOM protocol, is monitored using inverse epifluorescence microscopy (Figure 1c). The fit of the average nanosensor luminescence under modulated illuminations at a range of angular frequencies much higher than the camera's acquisition rate delivers a 2D projection of the nanosensor's phosphorescence lifetime 3D map within the agarose pad (Figure 1d).^[^ ^36^ ^]^ We assume that O_2_ uniformly diffuses throughout the sample in view of its fairly similar diffusion coefficient in most liquids.^[^ ^38^, ^39^ ^]^ Hence, we correct the experimental lifetime, τ_exp_, 2D map from both the photon collection function of the microscope and the O_2_‐concentration‐dependent variations in nanosensor brightness and phosphorescence lifetime. This provides a corrected nanosensor's phosphorescence lifetime (τ_cor_) map (Figure 1d), which yields the map of O_2_ concentration in the cell plane (Figure 1e). The latter map is eventually processed within a reaction‐diffusion framework to extract the O_2_ cellular flux by fitting the data accordingly (Figure 1e).

### 2.2 The O_2_ Nanosensor

The O_2_ nanosensor has been designed to exhibit functionality together with minimal invasiveness. Hence, for sensing, we adopted platinum‐octaethyl‐porphyrin (PtOEP). This widely used O_2_‐quenchable complex has benefited from thorough spectroscopic characterization.^[^ ^40^, ^41^ ^]^ In particular, it exhibits favorable spectroscopic features in absorption and emission, which enable us to excite it at 535 nm (where light absorption by the photosynthetic apparatus is minimal)^[^ ^42^ ^]^ and recover its luminescence at 640 nm (which limits interference from endogenous fluorescence from chlorophyll). It has been embedded in the hydrophobic core of a nanoparticle bearing a hydrophilic corona, which was conceived to ensure colloidal stability and lack of toxicity.

To produce the nanosensor, we nanoprecipitated a tetrahydrofuran (THF) solution of PtOEP, poly(styrene)‐graft‐poly(ethylene oxide) (PS‐g‐PEO), and bis(2‐ethylhexyl)sebacate (DOS) in water, which proved fast, easy, and reliable, involving commercially available reagents only. In a preliminary step (see Subsection S2.1, Supporting Information), we investigated how the molar absorption coefficient and brightness of the nanoparticles produced by nanoprecipitation of a PS‐g‐PEO solution in THF (10 g L^−1^) depended on the targeted concentrations of PtOEP and DOS over the 5−110 and 3−300 µM ranges, respectively. From this analysis, we identified 55 mM PtOEP and 34 µM DOS as optimal concentrations for producing high‐performance nanosensors.

**Figure 2:**
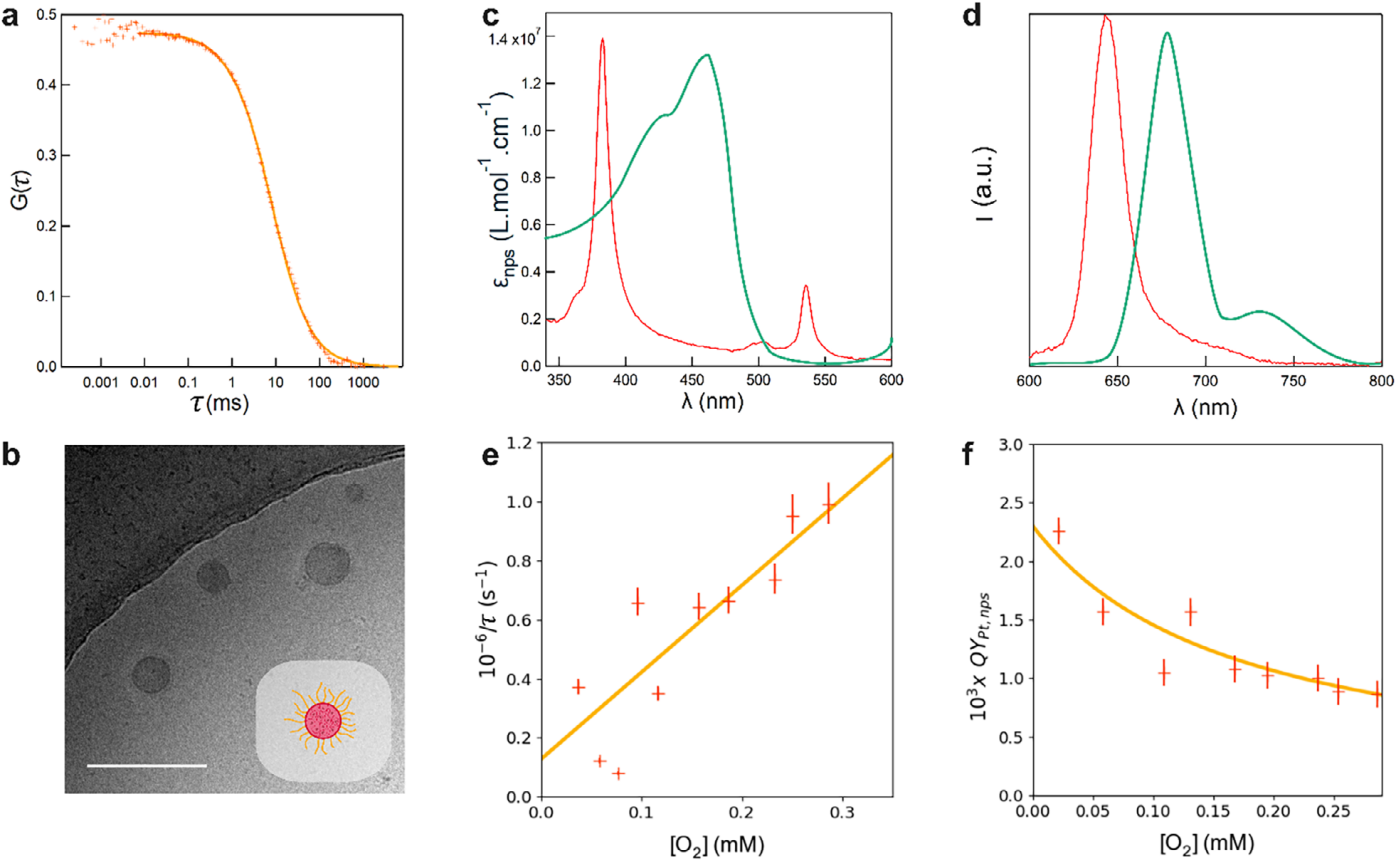
Characterization of the O_2_ nanosensor. a) Auto‐correlation function of the PtOEP luminescence emission. After fitting the experimental data (red markers) with Equation (S1, Supporting Information) (solid line), the diffusion time and concentration of the nanosensor were estimated as 8.3 ± 0.5 ms and 2.7 ± 0.1 nM, respectively; b) CryoTEM image. Scale bar: 200 nm. Insert: scheme of the hydrophobic PtOEP‐labeled polystyrene core and hydrophilic PEG corona of the nanosensor. Whereas the spherical polystyrene cores appear dark against the lighter amorphous ice background, the PEG coronae are not sufficiently contrasted to be visible; c,d) Absorption and luminescence emission d) spectra (red lines). The absorption and fluorescence spectra of chlorophyll a and b are also displayed in arbitrary units as green lines; e,f) Calibration of the dependence of the inverse of the luminescence lifetime τ and luminescence quantum yield Φ of the nanosensor on O_2_ concentration. Fits with Equations (S30 and S31, Supporting Information) (solid line) of the experimental data (cross markers) yield 1/τ(s) = 2.3 × 10^9^[O_2_](mol.L^−1^) + 1.3×10^5^ and Φ = 2.3 × 10^−3^/(1 + 5800[O_2_](mol L^−1^)). Solvent: water; *T* = 293 K.

We then investigated the structural and photophysical properties of the nanosensor. Fluorescence correlation spectroscopy (FCS) first revealed the production of luminescent nanoparticles at a concentration of 1 nM, with a mean hydrodynamic diameter of 170 nm (**Figure** **2**a), suggesting the PEG chains to be arranged in a brush regime (see Subsection S2.2, Supporting Information). The spherical polystyrene core of the nanoparticles was also observed with cryogenic transmission electron microscopy (CryoTEM), which evidenced diameters between 40 and 120 nm (Figure 2b).

The absorption spectrum of the nanosensor is displayed in Figure 2c. It exhibits main peaks at 380, 509, and 535 nm expected for PtOEP from literature data.^[^ ^41^ ^]^ They were further found to be similar in position and shape in THF (see Figure S8, Supporting Information). Thus, we used the molar absorption coefficient of PtOEP in the latter solvent (7.0 × 10^4^ mol^−1^ L cm^−1^) and the molar absorption coefficient of the nanosensor (3 × 10^8^ mol^−1^ L cm^−1^) to estimate that each nanosensor particle contained 4300 PtOEP complexes on average, corresponding to a PtOEP incorporation yield of 50% within the nanosensor during nanoprecipitation. Upon excitation at 535 nm, the nanosensor exhibits an emission spectrum with a 35 nm wide‐emission band centered at 644 nm (Figure 2d), in line with prior reports^[^ ^41^ ^]^ and the behavior observed in THF (see Figure S9, Supporting Information).

In aerated water, O_2_ efficiently quenches PtOEP luminescence, and the nanosensor exhibits a low 2.3 × 10^−3^ luminescence quantum yield and a main luminescence lifetime component of 1.5 ± 0.1 µs. In order to evaluate the nanosensor sensitivity toward O_2_ concentration, we implemented an experimental protocol exploiting sodium dithionite as an O_2_ scavenger (see Subsection S2.4, Supporting Information).^[^ ^43^ ^]^ Hence, we obtained calibration curves obeying the Stern–Volmer's law describing the O_2_ concentration dependence of both the inverse of the luminescence lifetime of the nanosensor and the luminescence quantum yield (Figure 2e,f).

### 2.3 The Sample of Microalgal Cells

With a cell diameter in the 10 µm range facilitating microscopy observation and as an established model organism for studies of photosynthesis and respiration, the freshwater algae *Chlamydomonas reinhardtii (C. reinhardtii)* are a favorable system to evaluate our approach to measure the O_2_ flux at the scale of single cells.^[^ ^44^ ^]^ We first confirmed that the presence of the O_2_ nanosensor did not generate any significant toxicity on the growth rate of cell populations (see Subsection S2.5, Supporting Information). Then, we produced the cell samples for microscopy observations. A dilute cell suspension of the CC‐4533 (CMJ030) strain in fresh Tris‐Acetate‐Phosphate (TAP) medium was deposited on a 200 µm‐thick agarose pad (1%w) containing the O_2_ nanosensor at 1 nM.

The sample was sealed and incubated for 4 h at 20 °C in the dark, which lowered its overall O_2_ concentration by promoting dark respiration of the cells. This preconditioning step increased both the brightness and luminescence lifetime of the nanosensor, which improved the signal‐to‐noise ratio of the RIOM‐based luminescence lifetime mapping. In a separate luminescence lifetime measurement performed on a batch of cells (see Subsection S2.6, Supporting Information), we evidenced that the O_2_ concentration dropped from 284 to 18 µM after 4 h in the dark. From this variation, we estimated the cellular dark respiration rate as *K* = –8.6 × 10^−15^ mol s^−1^ cell^−1^, consistent with previously reported values.^[^ ^45^, ^46^, ^47^, ^48^, ^49^ ^]^

### 2.4 Acquisition of the Spatial Profile of the Nanosensor Luminescence Lifetime Around Single Cells

We first briefly imaged the sealed sample with constant 480 nm light at an intensity of 580 µmol photons m*^−^* ^2^ s*^−^* ^1^ (14 mW cm^−2^) to select an isolated cell within the 160 × 160 µm^2^ field of view. Then, the sample was successively left in the dark for 5 min to recover from the brief 480 nm illumination and submitted to a 10‐s pulse of constant 480 nm light at the same intensity to reactivate the photosynthesis pathway.^[^ ^50^ ^]^ We then applied the RIOM protocol to acquire the map of the nanosensor's luminescence lifetime. For this purpose, we recorded a series of luminescence images with a 12 Hz acquisition frequency, when applying an illumination sequence alternating 1 s of sinusoidally modulated 540 nm light at an intensity of 7 × 10^−2^ mol photons m^−2^ s^−1^ (1.5 W cm^−2^) with 100% duty cycle and 1 s in darkness (to refresh a same initial state). This illumination pattern was repeated across ten logarithmically spaced modulation frequencies ranging from 800 Hz–8 kHz. The modulation durations (20 s) were chosen to exceed the expected O_2_ production response time (milliseconds^[^ ^51^ ^]^) and the time needed for establishing a stable O_2_ concentration profile (hundreds of milliseconds), as discussed in Section S3 (Supporting Information).

**Figure 3:**
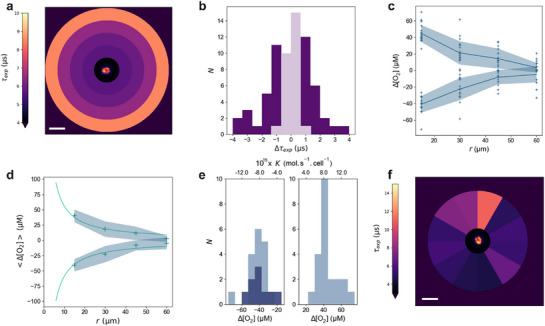
Concentration and flux of O_2_ at single cells. a) Map of the O_2_ nanosensor luminescence lifetime τ_exp_, averaged over concentric coronae centred on a single *C. reinhardtii* cell, as processed from the RIOM data. Central insert: 675 nm fluorescence image of the microalga. Scale bar: 20 µm. b) Histogram of the lifetime variation Δτ_exp_ = τ_exp_(*r* = 15 µm)–τ_exp_(*r* = 60 µm) over *N* = 69 single cells. Light purple: 27 cells for which Δτ_exp_ was found lower than the confidence interval (Δτ_exp_ < 1.4 µs); deep purple: 42 cells for which Δτ_exp_ was significant to extract an oxygen flux (Δτ_exp_ > 1.4 µs); c) Spatial profiles of the local O_2_ concentration deviation Δ[O_2_](r), defined relative to the mean concentration at *r* = 60 µm for the *N* = 42 cells exhibiting processable information. Solid line: Mean profiles for each type of subpopulation, exhibiting either dominant photosynthetic O_2_ production or respiratory O_2_ consumption. Light blue area: standard deviation over all the profiles from the same type calculated for each radius; d) <Δ[O_2_]> profiles averaged from c over the cell population of both categories to retrieve the mean O_2_ cellular outflux or influx. Markers: average experimental data for each profile type; light blue area: ± standard deviation; green line: fit with Equation (S38, Supporting Information); e) Histograms of Δ[O_2_] and the associated cellular O_2_ flux *K*, computed using Equation (S38, Supporting Information) for the *N* = 42 cells displayed in c (light blue) or *N* = 9 DCMU‐treated cells (deep blue); f) Map of the nanosensor luminescence lifetime τ_exp_ obtained from RIOM data under the assumption of anisotropic spatial O_2_ diffusion around the cells. Insert: Fluorescence image of the microalga collected at λ_em_ = 675 nm, showing strong endogenous fluorescence at a cell pole, corresponding to the chloroplast. Scale bar: 20 µm.

After masking the cell and its close area to eliminate any interference from endogenous fluorescence, the RIOM image recorded at each frequency was corrected from the mean background. Assuming isotropic O_2_ diffusion from the cell, we averaged the RIOM signal over concentric coronae of increasing radii *r* centered on the cell in order to increase the signal‐to‐noise ratio. The frequency‐dependence of the mean RIOM signals in each corona was then fitted^[^ ^36^ ^]^ to yield the experimental map of the nanosensor luminescence lifetime τ_exp_ (**Figure** **3**a). Figure 3b displays the histogram of lifetime change Δτ_exp_, defined as the difference in τ_exp_ between *r* = 15 µm and *r* = 60 µm, over 69 single cells. The distribution follows a bell‐shaped curve and it evidences both positive and negative variations, showing a diversity of O_2_ fluxes within the cell population.

### 2.5 Extraction of the O_2_ Flux from the Experimental Spatial Profile of the Nanosensor Luminescence Lifetime

To further analyze the data, we compared the variation in luminescence lifetime Δτ_exp_ to the confidence interval retrieved with the RIOM protocol (see Subsection S2.7, Supporting Information). For 27 single cells, Δτ_exp_ remained within the confidence interval, indicating that while the local O_2_ concentration could be probed, their O_2_ fluxes could not be reliably quantified. These cells are presumed to be near the compensation point between respiration and photosynthesis, likely reflecting an interrupted photosynthetic induction.^[^ ^52^ ^]^ In contrast, the spatial profile of the nanosensor luminescence lifetime for the remaining 42 cells allowed for quantification of the cellular O_2_ flux. We first noticed that the RIOM images collected with epifluorescence microscopy represent 2D projections of the RIOM signal, thus integrating the contribution of all the nanosensors present across the full thickness of the agarose pad. To correct for this projection effect, we modeled the theoretical 3D O_2_ concentration profile surrounding a microalgal cell deposited on the agarose pad (see Figure 1b; Section S3, Supporting Information). Taking into account the O_2_ concentration‐dependence of the nanosensor brightness and the photon collection function of the epifluorescence microscope (see Subsections S2.4 and S2.8, Supporting Information), we derived a correction factor to apply to τ_exp_(*r*), yielding the corrected spatial profile of the nanosensor luminescence lifetime in the cell plane, τ_cor_(*r*) (see Subsection S1.4.4, Supporting Information). Finally, we converted τ_cor_(*r*) into a spatial map of O_2_ concentration in the cell plane using the O_2_ concentration‐dependence of the nanosensor luminescence lifetime shown in Figure 2e.

Figure 3c displays Δ[O_2_](r), i.e., the difference of the O_2_ concentration at radius *r* in the cell plane from its mean value at 60 µm. We observed two categories of monotonously varying spatial profiles: positive Δ[O_2_] with a mean value of +41 ± 9 µM originated from cells with net O_2_ production, i.e., exhibiting dominant photosynthetic activity, while negative Δ[O_2_] with a mean ‐36 ± 8 µM value were assumed to originate from cells exhibiting dominant respiration.

The <Δ[O_2_]> profiles averaged over the cell population of both categories evidenced in Figure 3c were fitted with Equation (S38, Supporting Information) to retrieve the corresponding mean O_2_ cellular flux in the framework of a reaction‐diffusion model (Figure 3d; Section S3, Supporting Information). Importantly, we theoretically established that the precise description of the cell geometry did not significantly affect the value of the retrieved O_2_ cellular flux when analyzing the <Δ[O_2_]> profiles over the range of distances that we exploited. The respiration‐dominant single cells yielded a mean O_2_ consumption rate *K* = −(7.9 ± 1.7) × 10^−15^ mol s^−1^ cell^−1^, in agreement with both literature data^[^ ^45^, ^46^, ^47^, ^48^, ^49^ ^]^ and our own measurements on cell populations (see Subsection S2.6, Supporting Information). The photosynthetically active cells yielded a mean O_2_ production rate *K* = (7.5 ± 1.6) × 10^−15^ mol s^−1^ cell^−1^. Although ten times lower than the range reported in the literature at 500 µmol photons m^−2^ s^−1^ (≈10 mW cm^−2^) light intensity,^[^ ^45^, ^48^, ^53^ ^]^ the latter flux matched our *K* measurement in a cell population at the longest times in the dark (see Figure S14b, Supporting Information).

The individual <Δ[O_2_]> profiles were further processed in a similar way in order to build a histogram of the O_2_ cellular fluxes for each cell (Figure 3e). These results were compared with the behavior of cells which had been treated with 3‐(3,4‐dichlorophenyl)‐1,1‐dimethylurea (DCMU), a herbicide inhibiting photosynthesis. The cells exposed to DCMU exhibited exclusively negative Δ[O_2_] values, supporting the attribution of this behavior to cells with a dominant respiratory activity.

### 2.6 Evidence for Anisotropy of the O_2_ Concentration Map Around Single Cells

In photosynthetic cells, O_2_ plays opposing roles in the two major energy‐transforming pathways: it is produced during photosynthesis in the chloroplasts and consumed during mitochondrial respiration. Photosynthesis upconverts carbon dioxide (CO_2_) and water into glucose and O_2_ by using light energy, while respiration conversely harnesses O_2_‐mediated oxidation of glucose and liberates CO_2_ and water. Therefore, chloroplasts and mitochondria are expected to exhibit some O_2_‐mediated coupling. Such a coupling has been discussed for driving photosymbiosis in biological evolution^[^ ^54^, ^55^ ^]^ and it might contribute through natural selection to fixing the relative position of chloroplasts and mitochondria in photosynthetic cells.^[^ ^56^, ^57^ ^]^ More broadly, it may also be involved at another scale in the spatial self‐organization of heterotrophs and autotrophs within complex, multicellular populations such as microbial mats.^[^ ^58^, ^59^, ^60^ ^]^

In order to address potential O_2_‐mediated coupling, we processed the same set of RIOM data, this time assuming that the spatial O_2_ distribution around the cells was anisotropic. In order to benefit from a satisfactory signal‐to‐noise ratio, we averaged in this case the RIOM signal over sectors of equal area radiating from the cell. The frequency dependence of the averaged RIOM signals in each sector was then fitted to unravel angular variations in the nanosensor luminescence lifetime τ_exp_. Among the 69 single cells examined, 16 belonging to both respiration‐dominated or photosynthesis‐dominated categories exhibited a significant angular dependence of τ_exp_, indicative of anisotropic O_2_ distributions. Figure 3f displays a representative example from a cell oriented such that its main axis lies within the microscope's focal plane. Consistent with expectations, the highest lifetimes, indicating the lowest O_2_ concentration, were observed opposite to the chloroplast, coherent with O_2_ consumption and therefore compensation by mitochondria located at the flagellar base. This observation has been supported by numerical computations evidencing anisotropy in the profile of extracellular O_2_ concentration that would result from antagonist O_2_ fluxes at the chloroplast and mitochondria (see Section S3, Supporting Information).

## 3 Discussion

### 3.1 A Reaction‐Diffusion Framework for Measuring Consumption/Production Fluxes

Analytical chemistry offers a wealth of methods to measure chemical concentrations. In contrast, there is currently a lack of methods to quantify the fluxes of chemical reactions.^[^ ^61^, ^62^ ^]^ In this work, we illustrate how the map of a freely permeant molecule interpreted within a reaction‐diffusion framework can be used to retrieve quantitative information on its production/consumption flux down to the single cell level. Below, we successively discuss the scope and limitations of our implemented approach.

Sensing in cells is currently performed intracellularly after labeling the analyte or adding a probe whose signal is altered by interaction with the analyte.^[^ ^63^, ^64^ ^]^ Whereas this strategy yields the concentration of the analyte, it does not yield information on its flux. By contrast, our approach quantitatively images the extracellular concentration of a membrane‐permeant analyte over a characteristic distance comparable to the cell diameter,^[^ ^37^ ^]^ which provides direct access to its flux of intracellular origin. Advantageously, this approach does not necessitate any cell modification nor intracellular addition of a probe, which limits the risks of abnormal physiological behavior or toxicity. It also eliminates the challenge of localizing the probe within the cell, which often exhibits heterogeneous analyte concentrations.^[^ ^65^ ^]^ However, the requirement for analyte permeability limits the set of target molecules. Although this excludes many large or charged species, the potential target list goes beyond gases and includes many small molecules that can cross cell membranes.

The relevance of the present reaction‐diffusion framework for measuring the consumption/production flux of a freely permeant molecule necessitates to make assumptions on its diffusive properties in the gel pad. First, the gel has to be homogeneous, which was fulfilled here, considering the protocol for the gel preparation and its homogeneity observed in optical microscopy. Then, the diffusion coefficient of the targeted analyte in the gel has to be known or measured for retrieving the flux sought for. In the present study, O_2_ is a ≈0.1 nm‐long molecule, and the mesh size of the agarose gel is in the 100 nm range.^[^ ^66^ ^]^ Hence, further considering the error on the extraction of O_2_ concentration with the RIOM protocol, we adopted the value of the diffusion coefficient of O_2_ measured in water at 293 K, which is close to reported values of the dioxygen diffusion coefficient in various hydrogels.^[^ ^67^ ^]^ It is here noteworthy that the absence of specific information of O_2_ diffusivity at the cell surface is not detrimental to retrieve the cellular O_2_ flux. First, the flux is conservative, whatever the distance from the cell surface. Then, our numerical simulations demonstrated limited cell geometry‐dependence of the O_2_ concentration on the radial distance when retrieving the O_2_ flux from analyzing the O_2_ concentration in a 15–60 µm distance range from the cell surface (see Subsection S3.2, Supporting Information).

The reaction‐diffusion framework additionally imposes limitations on the temporal and spatial resolutions of this analytical approach. It assumes that the concentration map of the targeted analyte is steady. With a 10 µm‐sized cell, this state is reached in a second, which fixes the temporal resolution as well for monitoring dynamic changes in flux within the cell. We also evaluated the accessible level of spatial information on the analyte concentration. We first established the robustness of this approach by evidencing that the cell geometry does not significantly alter the value of the retrieved cellular flux of the analyte. In particular, we derived an analytical expression enabling end‐users to predict the spatial analyte concentration profile based on estimated flux, facilitating the choice of suitable reporters and imaging conditions. Finally, we reported preliminary experimental and theoretical results showing that our approach can also evidence an anisotropy of the intracellular metabolic flux.

### 3.2 Luminescence Lifetime to Access the Concentration Map of the Targeted Analyte

Although it is not necessary to implement the reaction‐diffusion framework for flux analysis, the luminescence lifetime observable benefits from several advantages for mapping the targeted analyte. First, its information is quantitative and does not depend on the concentration of the sensing luminophore. Then, when using phosphorescent sensors with long luminescence lifetimes that are currently available for key analytes such as dioxygen,^[^ ^12^, ^13^ ^]^ carbon dioxide,^[^ ^68^ ^]^ hydrogen sulfide,^[^ ^69^ ^]^ and other membrane‐permeant small molecules,^[^ ^69^ ^]^ the signal can be efficiently distinguished from short‐lived background autofluorescence—a particularly important benefit when working as here with photosynthetic organisms.

State‐of‐the‐art of phosphorescence lifetime imaging microscopy (PLIM^[^ ^70^ ^]^) relies on protocols of modulated illumination and image acquisition, which harness sophisticated and costly detectors endowed with high‐frequency modulation or fast gating.^[^ ^71^, ^72^, ^73^, ^74^ ^]^ In this study, we preferred to adopt the RIOM protocol.^[^ ^36^ ^]^ Indeed, from thoroughly exploiting the response of the phosphorescent probe photocycle to modulated illumination, this protocol achieves to deliver the map of phosphorescence lifetime not only with a much cheaper wide‐field imaging optical setup but also in a shorter time, which is here favorable due to the light sensitivity of the imaged sample.

### 3.3 Metabolic O_2_ Fluxes in Single Microalgal Cells

In this study, we retrieved the distribution of the metabolic O_2_ fluxes in single microalgal cells. Whereas our result on respiration‐dominant single cells was in line with the measurement on cell populations, photosynthetically active cells show around ten‐time lower O_2_ production rate than recorded in oxic batch samples.^[^ ^45^, ^48^, ^53^ ^]^ Our protocol imposes to keep the cells for four hours in darkness in a sealed chamber, in order to reach the low O_2_ concentration required for optimal function of the nanosensor. In these hypoxic dark conditions, cells experience a situation similar to the night in natural conditions, and fermentative pathways are activated.^[^ ^75^ ^]^ Reinduction of photosynthesis then takes longer than ten minutes,^[^ ^52^ ^]^ which is much longer than the total light received by the cells in our protocol. It is thus expected to find lower values than in oxic conditions used in other reports.

Interestingly, our experimental conditions generate a range of behaviors in the treated cells, from fully respiratory to lowly photosynthetically active. While we cannot fully exclude that the specific light and O_2_ conditions experienced by each cell could slightly vary, especially considering the time taken to select isolated cells, cell proximity, or cell orientation compared to light direction in the chamber, this cell‐to‐cell variation could also reflect metabolic diversity in our sample. Indeed, individual cells could show a range of rates in photosynthesis induction, which would explain the observed range of O_2_ fluxes after ten seconds of light.

In the specific case of O_2_ sensing in photosynthetic cells, confirming and extending these observations will necessitate a brighter nanosensor to improve signal‐to‐noise ratio, as well as enhanced control over sample preparation to get angular information on the cell orientation with respect to the focus plane.

## 4 Conclusion

This manuscript demonstrates the relevance of applying a reaction‐diffusion framework to retrieve information on the cellular flux of membrane‐permeant metabolites at the single cell level and on the scale of seconds. In this work, we specifically implemented the RIOM protocol to acquire wide‐field images of the phosphorescence lifetime of an O_2_‐sensitive nanosensor embedded in an agarose pad, on which individual cells of the freshwater alga (*C. reinhardtii)* were sparsely deposited. From these data, we extracted the spatial profiles of O_2_ concentration surrounding single cells, which were processed by fitting to determine the cellular O_2_ flux. The resulting distribution demonstrated that the cells from the same population can exhibit a wide range of photosynthesis‐to‐respiration activity ratios. We could further bring initial evidence for anisotropy in the spatial O_2_ concentration profile around individual cells, potentially reflecting the asymmetry in the positioning of the chloroplast and mitochondria in the microalgae.

## 5 Experimental Section

### Reagents

For the nanosensor production: Bis(2‐ethylhexyl)sebacate (DOS), tetrahydrofuran (THF), sodium dithionite (Na_2_S_2_O_4_), tris(hydroxymethyl)aminomethane (Tris), platinum octaethylporphyrin (PtOEP), rhodamine 6G (R590), and fluorescein were purchased from Sigma–Aldrich; Poly(styrene)‐graft‐poly(ethylene oxide) (PS‐g‐PEO) was obtained from Polymer Source, Inc (sample P43260‐SEOcomb). In this sample, poly(ethylene oxide) was hydroxy terminated (M*_n_* = 7000‐g‐4500 g mol^−1^). Polybead Carboxylate Microspheres 3, 4.50, 6, and 10 µm were from Polysciences.

### O_2_ Nanosensors

The O_2_ nanosensors were produced through nanoprecipitation. THF stock solutions of PtOEP (0.88 mM) and PS‐g‐PEO (10 g L*^−^* ^1^) were prepared and then stored at 4 °C. They were always sonicated 5 min before use.

General procedure. Appropriate volumes of PtOEP stock solution, *V_Pt_^stock^*, and pure DOS, *V_DOS_*, were mixed together with *V_pol_^stock^* = 160 µL of the PS‐g‐PEO stock solution. The resulting mixture was then quickly injected in *V_sol_* = 7 mL of deionized water, stirred at 1000 rpm in a 25 mL round‐bottomed flask. Argon was flown in the water‐THF mixture during 1 h in order to evaporate THF.^[^ ^76^ ^]^ The suspension was then dialyzed overnight in a 3–12 mL dialysis cassette (Slide‐A‐Lyzer, Thermo Scientific) with 3.5 K molecular weight cutoff (MWCO) immersed in deionized water. The solution was then collected and filtered through a 0.2 µm polytetrafluoroethylene (PTFE) filter (Acro 50 PTFE membrane, Sigma–Aldrich). The suspensions of nanoparticles were stored in sealed vials at 4 °C.

Production of the final O_2_ nanosensor. *V_Pt_^stock^* = 248 µL and *V_DOS_* = 2.6 µL were mixed together with *V_pol_^stock^* = 160 µL of the PS‐g‐PEO stock solution. The general procedure was then applied as above.

PtEOP‐loaded polystyrene microbeads. 1 mM PtOEP THF stock solution was prepared and stored at 4 °C. It was always sonicated for 5 min before use.

PtOEP stock solution (140 µL) was mixed at 500 rpm with 260 µL of ultrapure water and 100 µL of a 2.5% (w/w) polystyrene microbeads suspension. The resulting suspension was then dialyzed overnight in a 0.5–3 mL dialysis cassette (Slide A‐Lyzer, Thermo Scientific) with 3.5 K molecular weight cutoff (MWCO) immersed in ultrapure water. The suspensions of particles were stored in sealed vials at 4 °C.

### Algae

Production. Green algae *(C. reinhardtii*, strain: CC‐4533) were provided by Institut de Biologie Physico‐Chimique (IBPC). Algae precultures were grown on standard Tris‐Acetate‐Phosphate (TAP) agar medium and renewed every two weeks. For sensing experiments, algae suspension was prepared by inoculating 200 mL of TAP liquid medium in a culture flask. After 2 days of growth under constant illumination (50 µmol photons m*^−^* ^2^ s*^−^* ^1^; ≈1 mW cm^−2^) and agitation at 140 rpm, 5 mL of the culture was added to 100 mL of fresh TAP medium. This culture was grown for one day more under the same conditions and then used for sensing experiments. Cell growth was evaluated at this final step by cell counts with an automated cell counter (Beckman Coulter Z1‐D Particle Cell Counter Size Analyzer) or by measuring optical density. For DCMU experiments, DCMU was added in the culture up to a final concentration of 10 µM.

Samples for the batch experiments. Cells of *(C. reinhardtii)* grown as described above were concentrated by centrifugation and resuspended in fresh TAP medium at 3 × 10^6^ cells mL^−1^. 90 µL of this cell suspension and 35 µL of the nanoprecipitated sensor suspension (10*^−^* ^9^ M in objects) were then mixed, deposited in a 125 µL microchamber delimited with a 250 µm‐thick spacer (Gene Frames AB0578; Thermo Scientific) on a circular glass coverslip, and then capped with another circular coverslip. This sample was then left 4 h in the dark at 20 °C in order to promote dark respiration of the cells.

Samples for the single‐cell experiments. 20 µL of low‐gelling temperature agarose (from Sigma–Aldrich) at 1% (w/w) in the nanosensor suspension (*C_nps_^sol^* = 10*^−^* ^9^M) was deposited in a 125 µL microchamber delimited with a 250 µm‐thick spacer (Gene Frames AB0578; Thermo Scientific) on a circular glass coverslip. The microchamber was capped with a rectangular microscope slide. The microalgae grown as described above were centrifugated and resuspended in fresh TAP medium. After 15 min at 4 °C, the agarose became solid and the upper coverslip was removed. 100 µL of suspension of cells at 3 × 10^5^ cells mL^−1^ was deposited on and around the agarose pad. The sample was covered and protected from light for 10 min so that the microalgae had time to sediment on the pad. Then, a new coverslip was placed on the top to seal the sample. The as conditioned sample was then left 4 h at 20 °C in the darkness in order to promote dark respiration of the cells.

### Statistical Analysis

The behavior of 69 *(C. reinhardtii)* cells was investigated. The number of cells exhibiting each type of behavior was indicated at the appropriate positions in the text and figure captions. Extracted values as local oxygen concentrations or oxygen fluxes correspond to the mean values among cells exhibiting a similar behavior, and the error was the standard deviation over those cells.

The preprocessing of raw data in order to extract the oxygen fluxes, too extensive to be detailed here, was provided in great detail in Section S1.4.4 (Supporting Information). Such processing was performed using codes written in the programming language Python.

## Conflict of Interest

The authors declare no conflict of interest.

## Author Contributions

A. B., L. J., E. M., H. M., L. T., and X. X. performed Conceptualization; L. J. and H. M. performed methodology; H. M. and L. T. contributed in software development; A. B., L. J., E. M., H. M., L. T., and X. X. performed validation; L. J., H. M., L. T. performed Formal analysis; T. L. S., E. M., H. M., V. R., L. T., and T. V. performed Investigation; K. B., F. G., E. I., H. M., V. R., T. V., X. X. contributed in resources; L. J. and H. M. performed data curation; L. J. and H. M. Wrote–the original draft; all authors wrote–reviewed and edited the manuscript; L. J. and H. M. performed visualization; L. J. performed supervision; L. J. performed project administration; L. J. led the Funding acquisition.

## Supporting information



Supporting Information

## Data Availability

The data that support the findings of this study are available from the corresponding author upon reasonable request.
