# Supplementary material for: A Reaction‐Diffusion Frame for Accessing Metabolic O2 Fluxes in Single Microalgal Cells with Low‐Cost Wide‐Field Imaging of Nanosensor Luminescence Lifetime
Source: Adv Sci (Weinh). 2025 Aug 29;12(42):e10903. doi: 10.1002/advs.202510903 (PMC12622426; doi:10.1002/advs.202510903)
Supplement: Supplementary file 1 — Supporting Information [file ADVS-12-e10903-s001.pdf]

A reaction-diffusion frame for accessing metabolic O_2 fluxes in single microalgal cells with low-cost wide-field imaging of nanosensor luminescence lifetime

H. Merceron,[1,2,*] E. Israelievitch,[3] V. Rollot,[1] T. Villarubias,[1] X. Xie,[4], * T. Le Saux,[1] K. Benzerara,[2]
F. Guyot,[2] A. Boulouis,[3,*] E. Marie-Bègue,[1,*] L. Thouin,[1,*] L. Jullien[1,*]

[1]CPCV, Département de chimie, École normale supérieure,
PSL University, Sorbonne Université, CNRS, Paris, France
[2]Muséum National d'Histoire Naturelle, Sorbonne Université, UMR CNRS 7590,
Institut de Minéralogie, de Physique des Matériaux et de Cosmochimie (IMPMC), 4 Place Jussieu, 75005 Paris, France
[3]Chloroplast biology and light perception in microalgae, UMR7141,
CNRS, Sorbonne Université, Paris, France
[4]Department of Chemistry, The Hong-Kong University of Science and Technology,
Clear Water Bay, Kowloon, Hong Kong, China

*To whom correspondence should be addressed; E-mail:
helene.merceron@sorbonne-universite.fr,,
xiexj@ust.hk,
emmanuelle.marie@ens.psl.eu,
laurent.thouin@ens.psl.eu,
Ludovic.Jullien@ens.psl.eu.

August 4, 2025

Contents

1 Complements to Materials and Methods **4**
1.1 Instruments 4
1.1.1 UV/Vis absorption and fluorescence spectrometers 4
1.1.2 One-photon fluorescence correlation spectroscopy (FCS) 4
1.1.3 CryoTEM imaging 5
1.1.4 Measurement of luminescent lifetime with synchronous detection 5
1.1.5 Epifluorescence microscope 6
1.2 Methods 7
1.2.1 Extraction of the molar absorption coefficient and the brightness of the PtOEP-labelled nanoparticles 7

1.2.1.1 Molar absorption coefficient . . . 7
1.2.1.2 Brightness . . . 7
1.2.2 Measurements of light intensity . . . 8
1.2.2.1 Epifluorescence microscope . . . 8
1.2.2.2 Light for batches experiments . . . 8
1.2.2.3 Conversion of energy units . . . 9
1.2.3 Luminescence lifetime measurements . . . 9
1.2.3.1 On the batch sample . . . 9
1.2.3.2 At single cells . . . 9
1.2.3.3 RIOM acquisition . . . 9
1.2.4 From the RIOM images to the O_2 flux at a single cell . . . 9
1.2.4.1 Processing of the RIOM data . . . 9
1.2.4.2 Correction of the experimental spatial dependence of the luminescence lifetime of the nanosensor . . . 10
1.2.4.3 Fitting parameters . . . 14
1.2.4.4 Units for the O_2 flux K . . . 14
1.2.5 Assessing angular anisotropy of the O_2 concentration around a single cell . . . 14

2 Preliminary developments **15**
2.1 Optimization of the concentrations of PtOEP and DOS for the O_2 nanosensor . . . 15
2.1.1 Optimization of the PtOEP concentration . . . 15
2.1.2 Optimization of the DOS concentration . . . 16
2.2 Theoretical evaluation of the conformation of the hydrophilic polymer chains in the O_2 nanosensor . . . 18
2.3 Comparison of the PtOEP photophysical properties in the O_2 nanosensor and in THF . . . 19
2.3.1 Absorption properties . . . 19
2.3.2 Luminescence properties . . . 19
2.4 Dependence of the luminescence properties of the nanosensor on O_2 concentration . . . 22
2.4.1 Protocol . . . 22
2.4.2 Dependence of the brightness and luminescence lifetime of the nanosensor on O_2 concentration . 23
2.5 Impact of the interaction of the O_2 nanosensor with microalgae . . . 24
2.6 Measurement of the O_2 flux from a cell population . . . 24
2.7 Determination of the confidence interval of the lifetime measurement with the RIOM protocol . . . 26
2.8 Measurement of the function of the photon collection of the epifluorescence microscope . . . 27

3 Theoretical computation of 3D profiles of the O_2 concentration around a single cell **28**

3.1 Modeling . . . 28

3.2 Extraction of an analytic law for O_2 diffusion upon assuming axial symmetry of the O_2 concentration profile 28

3.2.1 Numerical simulations . . . 28

3.2.2 An analytic fitting function for the O_2 concentration profile . . . 30

3.3 Extraction of the O_2 concentration profile upon leveling off the assumption of axial symmetry . . . 32

3.3.1 Numerical simulations . . . 32

3.3.2 Results . . . 32

1 Complements to Materials and Methods

1.1 Instruments

1.1.1 UV/Vis absorption and fluorescence spectrometers

UV/Vis absorption spectra were recorded on a UV/Vis spectrophotometer (Cary 300 UV-Vis, Agilent Technologies, Santa Clara, CA) at 293 K equipped with a Peltier 1×1 thermostatic cell holder). Luminescence measurements were acquired on a LPS 220 spectrofluorometer (PTI, Monmouth Junction, NJ), equipped with a TLC50 cuvette holder (Quantum Northwest, Liberty Lake, WA) thermoregulated at 293 K, with samples exhibiting an absorbance lower than 0.05 to avoid inner filter effects.[1] Samples were contained in 1 cm × 0.2 cm (500 µL; the cuvette content was not stirred) quartz cuvettes (Hellma Optics, Jena, Germany).

1.1.2 One-photon fluorescence correlation spectroscopy (FCS)

The experiments were performed on a home-built one-photon epi-illumination set-up. The sample was excited at 488 nm with a laser diode (LBX-488, Oxxius, France) using a 60x objective (Olympus UPLSAPO 60X W NA 1.2, Evident, Tokyo, Japan). The fluorescence emission was collected with the same objective, reflected with a FF506-Di03 dichroic mirror, (IDEX Health & Science LLC, Rochester, NY) passed through an emission filter (FF507 - LP; IDEX Health & Science LLC), and was focused on the entrance of an optical fiber (50 µm diameter, M14L02, Thorlabs) using a $f = 200$ mm lens (AC254-200, Thorlabs). Light from the optical fiber was then split to allow simultaneous acquisition on two separate avalanche photo-diodes (SPCM-AQR-14 APD, Excelitas, Pittsburg, PA). For each run, the cross-correlation function $G(\tau)$ from the two avalanche photo-diodes signals was calculated by a LSI Correlator digital correlator (LSI, Fribourg, Switzerland). The data were recorded over 10 acquisitions of 30 s. The FCS measurement was performed on a drop of 15 μL of suspension of nanoparticles deposited on a glass surface at the center of the excitation laser beam. A 20 nM fluorescein solution (Sigma Aldrich) in 20 mM Tris pH=8 buffer (Sigma Aldrich) was used for calibration. Assuming the nanoparticles to be non-reactive and freely diffusing in a Gaussian light excitation volume, the correlation function $G(\tau)$ of the luminescence signal I obeys Eq.(S1)

$$G(\tau) = \frac{< I(\tau)\, I(t+\tau) >}{< I(t)^2 >} = 1 + \frac{1}{\overline{N}} \left(\frac{1}{1 + \frac{\tau}{\tau_{nps}}} \right) \tag{S1}$$

where $\overline{N}$ is the mean number of luminescent objects within the excitation volume, and τ_{nps} is the translational diffusion time of the nanoparticles.[2] Thus, the fit of the experimental correlation function with Eq.(S1) yields $\overline{N}$ and τ_{nps}. After calibration, by using fluorescein as a reference with a known diffusion coefficient ($D_{fluo} = 3.4 \times 10^{-10}$ m^2.s^{-1}[3]), τ_{nps} can be used to yield the diffusion coefficient D_{nps} of the nanoparticle with Eq.(S2)

$$D_{nps} = \frac{\tau_{fluo}\, D_{fluo}}{\tau_{nps}} \tag{S2}$$

where τ_{fluo} is the fluorescein diffusion time measured during calibration FCS experiments. The hydrodynamic diameter d_{nps} of the nanoparticles can then be extracted from the Stoke-Einstein relationship (S3) :

$$d_{nps} = 2 \, \frac{k_B \, T}{6\pi \, \eta_{sol} \, D_{nps}} \tag{S3}$$

where k_B is the Boltzmann constant, T is the absolute temperature, and η_{sol} is the solvent viscosity. On the other hand, the concentration of the nanoparticles in the suspension C_{nps}^{sol} can be retrieved from the intercept of the autocorrelation fonction G_{nps}^0 by further exploiting the intercept of the autocorrelation function, $G_{fluo}^0 = G_{fluo}(\tau = 0)$ obtained during the calibration with the fluorescein used at a known concentration.

$$C_{nps}^{sol} = \frac{C_{fluo} \, G_{fluo}^0}{G_{nps}^0}. \tag{S4}$$

1.1.3 CryoTEM imaging

The nanoparticle samples were analysed by CryoTEM with a TEM Jeol 2100 equipped with a thermionic emission gun (LaB6), a cryo-polar piece (0.27 nm), a dedicated detector for bright field scanning imaging (STEM-BF), a Gatan US 1000 (2k × 2k) type U sensitive camera, and a cryoTEM holder (90 K). The samples were placed on a 3 mm diameter Lacey carbon film on Copper grid.

1.1.4 Measurement of luminescent lifetime with synchronous detection

The luminescence lifetime of the PtOEP-labeled nanoparticles was measured with lock-in detection either on a home-built trans-illumination set-up (for calibrating the dependence of the luminescence lifetime of the nanosensor on O_2 concentration; see subsection 2.4) or on the epifluorescence microscope described below (for measuring the O_2 production by an algae batch, see subsection 2.6, and for RIOM validation, see subsection 2.7).

In the trans-illumination set-up, the sample was excited at 450 nm with a laser diode (PL450B, Thorlabs, NJ), the amplitude of which was modulated with a sinusoidal signal from 1 kHz to 10 MHz. The luminescence emission was passed through an emission filter (FL670-10, Thorlabs, NJ), and collected by a photomultiplier tube (H10492-002, Hamamatsu). The modulation of the luminescence emission was measured by synchronous detection with a lock-in amplifier (HF2LI 50 MHz, Zurich instruments).

The dependence of the amplitude of the luminescence modulation on the frequency f of light excitation is related to the lifetime τ of the excited state by Eq.(S5)

$$M(f) = \frac{a}{\sqrt{1 + (2\pi f \tau)^2}} \tag{S5}$$

when the sample exhibits a single lifetime for the excited state.[1]

When the sample exhibited a more complex behavior, we considered that it exhibited two different lifetimes τ_1 and τ_2. Then we processed the dependence of the amplitude of the luminescence modulation on the frequency f of light

excitation with Eq.(S6)

$$M(f) = \frac{a_1}{\sqrt{1 + (2\pi f \tau_1)^2}} + \frac{a_2}{\sqrt{1 + (2\pi f \tau_2)^2}}. \tag{S6}$$

1.1.5 Epifluorescence microscope

A diagram of the components of the home-built inverted epifluorescence microscope is shown in Figure S1.

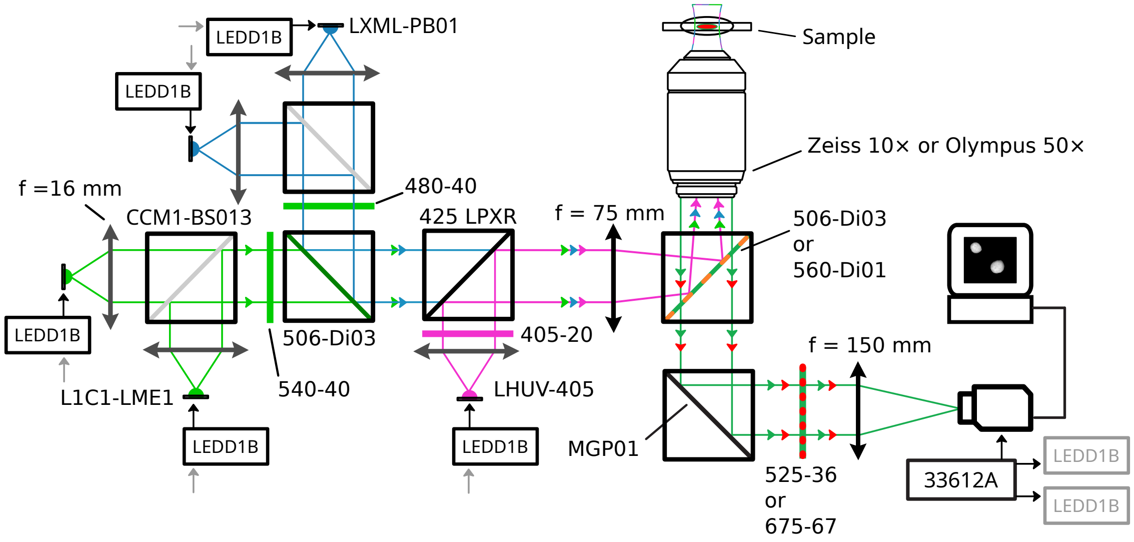

Figure S1: *Diagram of the components of the home-built fluorescence microscope.*

The setup is equipped with several light sources: a 405 nm LED (LHUV-0405 LED, Lumileds, San Jose, CA) , two blue LEDs (470 nm, LXZ1-PB01, Lumileds) and two green LEDs (540 nm, L1C1-LME1000000000 LED, Lumileds). Each LED is supplied by a LED driver (LEDD1B, Thorlabs, Newton, NJ), a pair of which can be modulated with a desired phase shift and frequency using a waveform generator (33612A, Keysight Technologies, Santa Rosa, CA). The light from each LED is collimated using a high numerical aperture condenser lens (ACL25416U, Thorlabs Inc., Newton, NJ; f = 16 mm). For the blue and green LED pairs, the collimated light beams from each LED are first combined using a 50:50 beam splitter (CCM1-BS013, Thorlabs) before being filtered with a corresponding excitation filter (HQ480/40, Chroma Technology Corp., Bellows Falls, VT, US for the blue LEDs or ET540/40, Chroma Technology Corp., for the greens LEDs). The two resulting light beams are then combined with a FF506-Di03 dichroic mirror (IDEX Health & Science LLC, Rochester, NY) into a single beam which passes though a second dichroic mirror (T425LPXR, Chroma Technology Corp., Bellows Falls, VT, US) to mix with the collimated light beam coming from the UV LED filtered using a ZET405/20X bandpass filter (Chroma Technology Corp.). A f = 75 mm (AC254-075-A, Thorlabs) is used to focus light at the back focal plane of a 10× (Fluar, Zeiss, Jena, DE; N.A. 0.5) or a 50× (MPlanFLN, Olympus Corp., Tokyo, JP; N.A. 0.8) objective after being reflected by a dichroic filter which is either a FF506-Di03 (IDEX Health & Science LLC, Rochester, NY) for Dronpa-2 containing samples or a FF560-Di01 (IDEX Health & Science LLC) for phosphorescent

dye-loaded objects. Fluorescence emission was collected with the imaging objective, filtered by a band pass filter, FF01-525/30 (IDEX Health & Science LLC) for Dronpa-2 containing samples or FF01-675-67 (IDEX Health & Science LLC) for phosphorescent dye-loaded objects, and reflected by a MPG01-350-700 (IDEX Health & Science LLC) mirror before being refocused onto the sensor of a iXon 897 EMCCD camera (Andor Technology, Belfast, UK) by a f = 150 mm lens (ACA254-150-A, Thorlabs). The trigger of the camera acquisition was synchronized with the start of the periodic excitation light using the option "External start" in the Solis software used to capture the images (Andor Technology).

1.2 Methods

1.2.1 Extraction of the molar absorption coefficient and the brightness of the PtOEP-labelled nanoparticles

1.2.1.1 Molar absorption coefficient The absorption spectrum of the aqueous solution of the PtOEP-labelled nanoparticles, A, was first recorded under aerated conditions (in the presence of O_2) between 300 and 800 nm. The contribution of light diffusion was then subtracted from the recorded spectrum by adopting a polynomial function for the baseline. The molar absorption coefficient of the PtOEP-labelled nanoparticles, ϵ_{nps}, was subsequently determined by using the Beer-Lambert's law given in Eq.(S7)

$$\epsilon_{nps} = \frac{A}{\ell C_{nps}^{sol}} \tag{S7}$$

where ℓ and and C_{nps}^{sol} designate the optical path length and the concentration of nanoparticles retrieved from the FCS experiments with Eq.(S4).

1.2.1.2 Brightness The excitation wavelength was set at 535 nm and the steady luminescence emission spectrum was recorded from 560 to 800 nm. The latter luminescence spectra were corrected by retrieving the corresponding spectrum recorded from the solvent.

The luminescence quantum yields $QY_{Pt,nps}$ were calculated using the corrected emission spectra of the sample and the standard rhodamine 6G[i] with Eq.(S8)

$$QY_{Pt,nps} = \frac{f_{6G}^{\lambda}}{f_{Pt}^{\lambda}} \frac{n_{H_2O}^2}{n_{EtOH}^2} \frac{\int_0^\infty I_{Pt}(\lambda_{ex}, \lambda_{em}) \mathrm{d}\lambda_{em}}{\int_0^\infty I_{6G}(\lambda_{ex}, \lambda_{em}) \mathrm{d}\lambda_{em}} QY_{6G}^{EtOH} \tag{S8}$$

with $f^{\lambda} = 1 - 10^{-A_{\lambda}}$ the absorption factor or the sample, f_{Pt}^{λ}, and of the standard, f_{6G}^{λ}, n_{sol} the refractive index of the solvent, and $\int_0^\infty I(\lambda_{ex}, \lambda_{em}) \mathrm{d}\lambda_{em}$ the integral of the emission spectrum of the sample and of the standard.[1]

The brightness of the PtOEP-labeled nanoparticles, B_{nps}^{λ}, is then defined by Eq.(S9)

$$B_{nps} = \epsilon_{nps}\, QY_{Pt,nps} \tag{S9}$$

[i] High fluorescence quantum yield determined with absolute measurements ($QY_{R590}^{EtOH} = 0.95 \pm 0.015$ in EtOH)[4],[5] at 500 nm excitation wavelength, close to the 535 nm wavelength presently used.

1.2.2 Measurements of light intensity

In this work, the light intensities have been measured either by using a power meter or through exploiting the fluorescent protein Dronpa-2 as a fluorescent actinometer together with 7-Hydroxy-9H-(1,3-dichloro-9,9-dimethylacridin-2-one) acting as a light intensity transferring fluorophore.[6]

1.2.2.1 Epifluorescence microscope In epifluorescence microscopy, the light intensity applied at 470 nm was directly measured from analyzing the time evolution of the fluorescence decay of an illuminated 35 μM Dronpa-2 solution in PBS (pH 7.4, 50 mM sodium phosphate, 150 mM NaCl) contained in a measurement chamber similar to the one used with the microalgae. When imaging the full field of view, our camera could not reach high enough an acquisition frequency to reliably retrieve the decay of the Dronpa-2 fluorescence upon illumination at the exploited light intensities. Therefore, we reduced the field of view to an area of 64×64 pixels^2. Hence, we could now achieve recording the fluorescence decay at 196 Hz upon turning on constant 470 nm light and then turning on constant 405 nm light over the 470 nm background.

Once the light intensity at 470 nm was known for all the experimental conditions, the light intensity at 540 nm was measured according to the protocol described in[6] by recording the level of fluorescence emission of a 9 μM solution of 7-Hydroxy-9H-(1,3-dichloro-9,9-dimethylacridin-2-one) acting as a light intensity transferring fluorophore from excitation at 470 nm to excitation at 540 nm.

1.2.2.2 Light for batches experiments The light intensity of the broad white halogene light source that was used for illuminating the batch of cells in the batch experiments was assessed with a powermeter upon considering that its maximum visible emission was at 685 nm. The spectra was established with the 3DFiberSpectrometer from GaudiLabs.

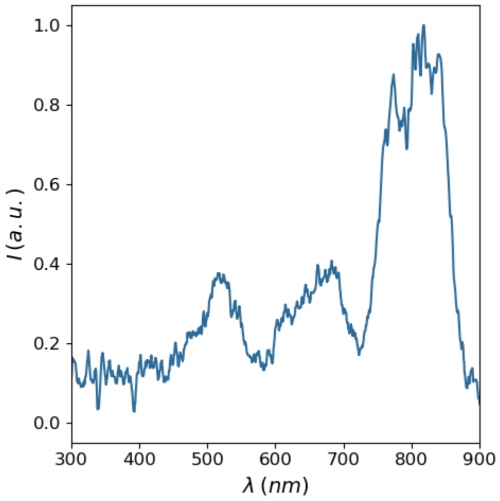

Figure S2: *Emission spectrum of the halogen lamp used for the batch experiments.*

1.2.2.3 Conversion of energy units

In this manuscript, the light intensities are provided in mol photons.m^{-2}.s^{-1}. This unit is currently used in actinometry. However, it is not often used in other fields such as optical microscopy, in which the researchers prefer to adopt W.m^{-2}. We provide below the conversion between both units.

We consider a monochromatic light of wavelength λ_{exc}. Its values in mol photons.m^{-2}.s^{-1} and W.m^{-2} are respectively denoted as $I(\lambda_{\text{exc}}, \text{mol photons.m}^{-2}.\text{s}^{-1})$ and $I(\lambda_{\text{exc}}, \text{W.m}^{-2})$. The relation between $I(\lambda_{\text{exc}}, \text{mol photons.m}^{-2}.\text{s}^{-1})$ and $I(\lambda_{\text{exc}}, \text{W.m}^{-2})$ is given in Eq.(S10)

$$I(\lambda_{\text{exc}}, \text{W.m}^{-2}) = \frac{hcN_A}{\lambda_{\text{exc}}} \times I(\lambda_{\text{exc}}, \text{mol photons.m}^{-2}.\text{s}^{-1}) \approx 0.12 \times \frac{\text{I}(\lambda_{\text{exc}}, \text{mol photons.m}^{-2}.\text{s}^{-1})}{\lambda_{\text{exc}}\,(\text{m})} \quad \text{(S10)}$$

with the Planck constant $h = 6.63\ 10^{-34}$ m^2.kg.s^{-1}, speed of light in a vacuum $c = 3.00\ 10^8$ m.s^{-1}, the Avogadro number $N_A = 6.02\ 10^{23}$ mol^{-1}, and where λ_{exc} is in m.

1.2.3 Luminescence lifetime measurements

1.2.3.1 On the batch sample

The measurement cell left for 4 h in the darkness was illuminated with a broad white lamp at light intensity I = 50 μmol photons.m^{-2}.s^{-1}. The luminescence lifetime of the O_2 nanosensor was measured every 30 min with lock-in detection.

1.2.3.2 At single cells

The measurement cell left for 4 h in the darkness was placed on the microscope stage, side with deposited cells facing the objective. Cell recognition and focus were performed at 480 nm excitation wavelength at 580 μmol photons.m^{-2}.s^{-1} light intensity. The sample was successively left in the dark for 5 min to recover from brief 480 nm illumination, and then submitted to a 10 s 480 nm constant illumination at 580 μmol photons.m^{-2}.s^{-1} to reactivate the photosynthesis pathway.

1.2.3.3 RIOM acquisition

The RIOM acquisition sequence was obtained by repeating 1 s sinusoidally modulated illumination at 540 nm at 7.10^{-2} mol photons.m^{-2}.s^{-1}light intensity with 100 $\%$ duty cycle, and then 1 s without illumination at 10 frequencies logarithmically spaced between 800 and 8 kHz. The camera was triggered at the beginning of illumination and a movie was recorded during the whole illuminating sequence.

1.2.4 From the RIOM images to the O_2 flux at a single cell

1.2.4.1 Processing of the RIOM data

The first step of the work flow associated to the processing of the RIOM data is displayed in Figure S3. The immediate area near to the cell was first masked in the RIOM image recorded at each frequency since we estimated that it could locally experience interference from endogenous fluorescence of the microalgae that could be mediated by light scattering through the pad. The mean background RIOM signal was subsequently established using a threshold, and was subtracted from the RIOM image at each pixel.

In order to retrieve the spatial dependence of the luminescence lifetime of the nanosensor (Figure S4a), we first assumed the distribution of the O_2 concentration around a cell to be isotropic. Hence, the field of view was first separated

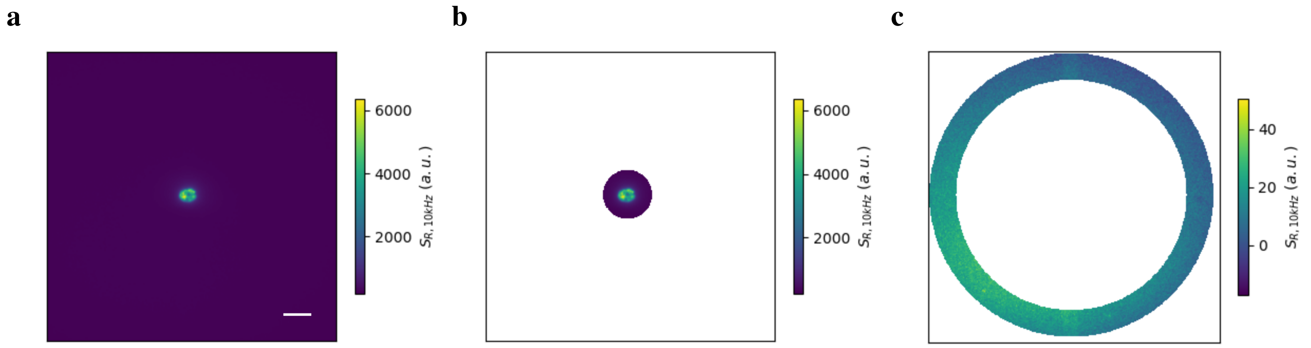

Figure S3: *Work flow for processing the 12 Hz-acquired image of the RIOM signal of a single cell in a sample submitted to modulated illumination at 10 kHz.* **a** : Initial RIOM image. Scale bar: 10 μm; **b**: Mask applied to avoid interference of light scattering from the microalgae cell with the luminescence response of the nanosensor; **c** : Mask evidencing the outer corona (r= 60 μm) over which the RIOM signal was averaged for further processing.

in concentric corona of increasing radii r centered on the cell, and the RIOM signal was then averaged over each corona. The dependence of the averaged RIOM signal at each corona on the frequency of light modulation was fitted with a simplified cut-off function (S11)

$$S_{RIOM}(f) = \frac{a}{1+(2\pi f\tau)^2} + b \tag{S11}$$

that enabled us to get better convergence with noisier data than the previously reported one.[7] Eventually, we could retrieve the spatial dependence of the luminescence lifetime of the nanosensor.

At that step, the experimental spatial dependence of the luminescence lifetime of the nanosensor around each investigated cell was extracted. To further analyze the data, we assessed the amplitude of the luminescence lifetime variation over the examined distance: $\Delta\tau_{exp} = \tau_{exp}(r_{min}) - \tau_{exp}(r_{max})$. When it was lower than the confidence interval of the luminescence lifetime with the RIOM protocol evaluated in subsection 2.7 ($\Delta\tau_{exp} < 2\sigma$), we could not reliably process the data to retrieve a meaningful value of the O_2 flux. Hence, only the cells that yielded $\Delta\tau_{exp} > 2\sigma$ were subsequently used for further processing.

1.2.4.2 Correction of the experimental spatial dependence of the luminescence lifetime of the nanosensor The RIOM images $S_R(r)$ collected with our epifluorescence microscope are two-dimensional projections of the RIOM signal $S_R(r,z)$ integrating the contribution of all the nanosensors along the optical axis z. Hence, the experimental spatial dependence of the luminescence lifetime of the nanosensor has to be corrected before applying Eq.(S32) for retrieving the spatial dependence of the O_2 concentration around each studied cell.

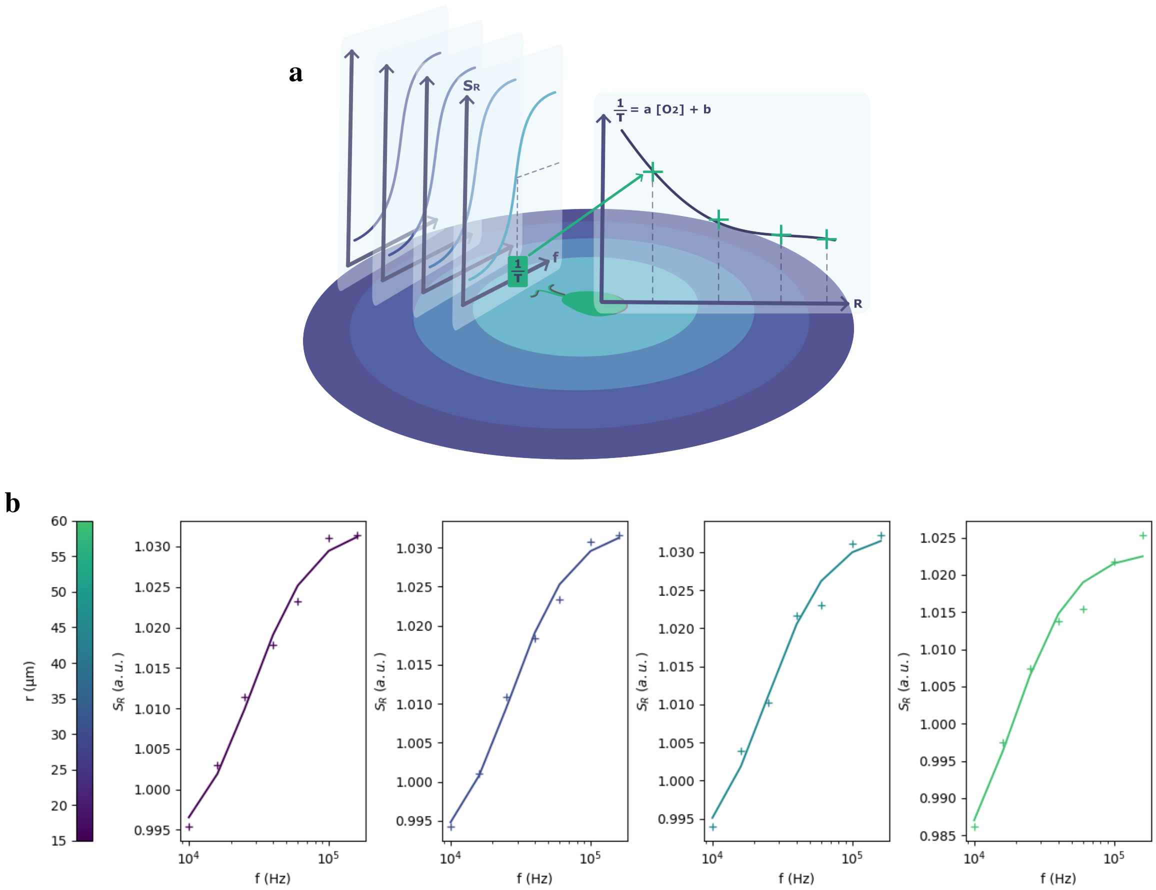

Figure S4: *Extraction of the spatial dependence of the luminescence lifetime of the nanosensor upon assuming an isotropic distribution of the O_2 concentration around a cell.* **a**: Principle; **b**: Experimental frequency-dependence of the RIOM signal averaged over each corona of radius r (from left to right: r = 15, 30, 45, 60 μm). Cross markers: experimental points; solid line: fit with Eq.(S11). Fitting parameters given in Table S1.

To access the factor to be applied to correct the experimentally measured luminescence lifetime $\tau_{exp}(r)$, we first generated the expected theoretical three dimensional profile of O_2 concentration around a microalgal cell within the agarose pad by using Eq.(S39) (Figure S5a), which assimilates the cell to an hemisphere on the coverslip acting as an impermeable surface (see Section 3). To compute this theoretical profile, we preliminarily assessed an order of magnitude for the O_2 flux K based on our experimental results. We evaluated the mean variation in O_2 concentration $\Delta[O_2]$ = $[O_2](r_{max})$-$[O_2](r_{min})$, with $[O_2](r)$ computed with Eq.(S32) and $\tau_{exp}(r)$. Hence, we could then extract an estimate of $K = 1 \times 10^{-14}$ mol.s^{-1}.cell^{-1} by using Eq.(S39), which subsequently proved to be in the right range after complete data processing (final retrieved value: $K = 7.5 \times 10^{-15}$ mol.s^{-1}.cell^{-1}). We then harnessed Eq.(S32) to compute the expected three dimensional profile of the luminescence lifetime of the nanosensor contained within the agarose pad (Fig. S5b).

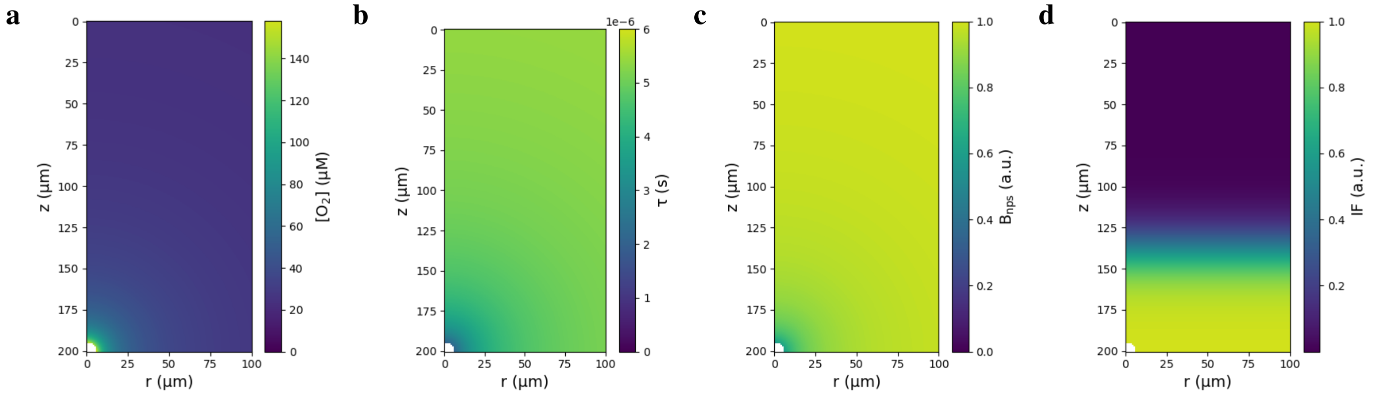

Figure S5: *From the experimental to the corrected luminescence lifetime of the nanosensor.* **a**: Theoretical three dimensional profile of O_2 concentration around a microalgal cell within the agarose pad as computed with Eq. (S39) by using $K_{out} = 1 \times 10^{-14}$ mol.s^{-1}, $\mathrm{D_{O_2}} = 2 \times 10^{-9}$ m^2.s^{-1}; **b**: Resulting theoretical three dimensional profile of the luminescence lifetime of the nanosensor within the agarose pad as computed with Eq. (S32); **c**: Resulting theoretical three dimensional profile of the brightness of the nanosensor within the agarose pad as computed with Eq. (S33); **d**: Photon collection function of the epifluorescence of the microscope as computed from Eq.(S36).

We eventually built the two dimensional projection, $\tau_{2D}(r)$ of the three dimensional profile of the luminescence lifetime of the nanosensor $\tau_{3D}(r, z)$ contained within the agarose pad by taking into account the O_2 concentration dependence of the nanosensor brightness ($B_{nps}(r, z)$, Figure S5c) and the photon collection function of our epifluorescence of the microscope ($IF(r, z)$, Figure S5d). The expression of $\tau_{2D}(r)$ is given in Eq. (S12).

$$\tau_{2D}(r) = \int_{z=0}^{z_{pad}} B_{nps}(r, z) \times IF(z) \times \tau_{3D}(r, z)\, dz \tag{S12}$$

To experimentally retrieve the cellular O_2 flux K_{out} finally sought for, we exploited the dependence of the O_2 concentration on the radius r from the cell when it is in the focal plane of the epifluorescence microscope. Hence, in order to access this information, we computed the relation (S13) existing between $\tau_{2D}(r)$ and the r-dependence of the luminescence lifetime occurring in the focal plane $\tau_{3D}(r, z = 0)$, which is denoted τ_{cor} in the Main Text for simplifying the notation.

$$\Psi(r) = \frac{\tau_{3D}(r, z = 0)}{\tau_{2D}(r)} \tag{S13}$$

Hence, we could establish the r-dependence of the correction factor $\Psi_{+O_2}(r)$ (see Figure S6).

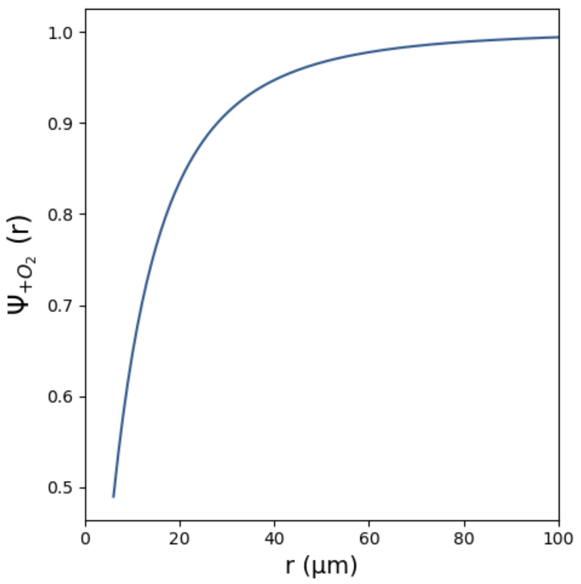

Figure S6: *Dependence of the correction factor $\Psi_{+O_2}(r)$ on the distance to the microalgae r to be applied to extract the cellular O_2 flux K_{out} from the spatial profile of luminescence lifetime $\tau_R(r)$ retrieved with the RIOM imaging protocol.*

The correction reported above and displayed in Figure S6 applies in the case of O_2 production. Considering the symmetry of the problem, in the case of O_2 consumption, the correction factor becomes

$$\Psi_{-O_2}(r) = \frac{1}{\Psi_{+O_2}(r)} \tag{S14}$$

For processing the RIOM data, the profiles of luminescence lifetime have been processed by comparing $\Delta\tau_{\text{exp}} = \tau_{\text{exp}}(r_{min}) - \tau_{\text{exp}}(r_{max})$ to the confidence interval σ of the luminescence lifetime retrieved with the RIOM protocol (see subsection 2.7):

1. if $\Delta\tau_{\text{exp}} < 2\sigma$: the r-dependence of the profile of luminescence lifetime is not significant regarding the uncertainty of the measurement. Only a global O_2 concentration can be extracted around the alga but without information on its spatial dependence.

2. if $\Delta\tau_{\text{exp}} > 2\sigma$: the r-dependence of the profile of luminescence lifetime is significant regarding the uncertainty of the measurement. Each experiment has then been unsupervisedly sorted in order to apply the right correction coefficient with the following condition:

   (a) if $\Delta\tau_{\text{exp}} > 0 : \tau_{3D}(r, z = 0) = \tau_{corr} = \Psi_{-O_2}(r) \times \tau_{exp}(r)$

   (b) if $\Delta\tau_{\text{exp}} < 0 : \tau_{3D}(r, z = 0) = \tau_{corr} = \Psi_{+O_2}(r) \times \tau_{exp}(r)$

Eventually, the r-dependence of $\tau_{3D}(r, z = 0)$ was fitted with Eq.(S32) to extract the O_2 flux K_{out} at each cell.

1.2.4.3 Fitting parameters

The fitting parameters used to retrieve the luminescence lifetimes with Eqs.(S11,S5) are reported in Table (S1).

Table S1: Fitting parameters used in the RIOM images in the Figures of the Main Text and the Supporting Information.

Figure	Fitting function	Amplitude	Offset	τ (ms)
1.2.4.2a	Eq.(S11)	$-4.0.10^{-2}$	1.0	$5.7.10^{-6}$
1.2.4.2b	Eq.(S11)	$-4.2.10^{-2}$	1.0	$5.9.10^{-6}$
1.2.4.2c	Eq.(S11)	$-4.4.10^{-2}$	1.0	$6.5.10^{-6}$
1.2.4.2d	Eq.(S11)	$-4.6.10^{-2}$	1.0	$8.5.10^{-6}$
S15a	Eq.(S5)	$6.8.10^{-4}$	$2.4.10^{-4}$	$5.6.10^{-6}$
S15b	Eq.(S11)	$-4.1.10^{-2}$	1,0	$4.8.10^{-6}$
S11a	Eq.(S5)	0.23	0.80	$2.7.10^{-7}$
S11b	Eq.(S6)	0.11 ; 0.67	0.81	$1.0.10^{-5}$; $1.5.10^{-6}$

1.2.4.4 Units for the O_2 flux K

Most O_2 cellular fluxes reported in the literature are expressed in mol.g(chl$_a$)$^{-1}$.h^{-1}, the molar flux reported to the chlorophyll massic content. Hence, we established the relation existing between O_2 fluxes in the latter unit and in mol.s^{-1}.cell^{-1} that we used in our work.

The specific absorption coefficient of chlorophyll a in acetone reported in the literature is $\alpha_{chl_a} = 88$ L.g^{-1}.cm^{-1}.[8] The difference between the absorption spectra of chlorophyll a between organic solvents and *in vivo* has also been computed: $\alpha_{chl_a,vivo} = 1.14 \times \alpha_{chl_a,acetone}$.[9] Eventually, we exploited the dependence of the absorbance at 685 nm of a *Chlamydomonas reinhardtii* culture on the cell concentration (see subsection 2.5) in order to extract the absorption coefficient per cell, $\alpha_{cell} = 5.1$ L.cell^{-1}. We could eventually compute the massic content of chlorophyll a, m_{chl_a} per cell

$$m_{chl_a} = \frac{\alpha_{cell}}{\alpha_{chl_a,vivo}} \quad \text{(S15)}$$

$$m_{chl_a} = 5.0.10^{-9}\ g(chl_a).cell^{-1} \quad \text{(S16)}$$

which enabled us to easily convert the O_2 cellular fluxes from one unit to another.

1.2.5 Assessing angular anisotropy of the O_2 concentration around a single cell

The same RIOM data were processed differently in order to assess the spatial anisotropy of the O_2 concentration around an alga cell. As above, the immediate area near to the cell was masked in each RIOM image to avoid interference from endogenous fluorescence of the microalga and the mean background RIOM signal was retrieved for each pixel using a threshold. In contrast, the field of view was now divided in sectors of identical area around the cell and the RIOM signal was averaged over each of them. The dependence of the averaged RIOM signal at each sector on the frequency of light modulation was fitted with Eq. (S11). Hence, we could then retrieve the angular dependence of the luminescent lifetime of the nanosensor around the alga.

2 Preliminary developments

2.1 Optimization of the concentrations of PtOEP and DOS for the O_2 nanosensor

2.1.1 Optimization of the PtOEP concentration

In a purpose of finding optimal conditions for sensing, we first produced nanoparticles at targeted PtOEP concentration $C_{Pt,nps}^{targ}$ ranging from 5 to 110 mM while keeping constant the targeted DOS concentration equal to 34 μM. Beyond adopting $V_{DOS} = 2.6$ μL, we fixed V_{Pt}^{stock} in the general procedure reported in the Main Text by considering that the yields of polymer nanoprecipitation and PtOEP incorporation within the nanoparticles were equal to 1, the nanoparticles were spherical and monodispersed, and the total volume of DOS and polymer injected in THF, $V_{pol,DOS}^{tot}$, was equal to the volume of the produced nanoparticles.[ii] Table S2 displays the results to target PtOEP concentration ranging from 5 to 110 mM.

$C_{Pt,nps}^{targ}$ (mM)	V_{Pt}^{targ} (μL)	PtOEP Incorporation yield (%)
5	22	76
10	45	33
15	67	82
25	113	61
35	157	36
55	248	76
75	337	42
110	498	34

Table S2: Targeted PtOEP concentration $C_{Pt,nps}^{targ}$ and corresponding volume of PtOEP stock solution V_{Pt}^{targ} used in the formulation.

We recorded the absorption and emission spectra as well as the FCS autocorrelation function of the luminescence signal of the different nanoparticle suspensions. Hence, we could retrieve the molar absorption coefficient, ϵ_{nps}, the brightness, B_{nps}, the diameter, and the concentration, C_{nps}^{sol}, of the corresponding nanoparticles. The mean number of PtOEP complex per nanoparticle, N_{Pt}, was then calculated with Eq.(S19) by using the molar absorption coefficient of the platinum complex measured in THF, ϵ_{THF}, which was supposed to be similar in THF and in the PS core of the nanoparticles.

$$N_{Pt} = \frac{\epsilon_{nps}}{\epsilon_{THF}} \tag{S19}$$

[ii] Then

$$C_{Pt,nps}^{targ} \approx \frac{C_{Pt}^{stock}\, V_{Pt}^{stock}\, \rho_{pol}}{V_{DOS}\, \rho_{pol} + Cm_{pol}^{stock}\, V_{pol}^{stock}} \tag{S17}$$

where ρ_{pol}, Cm_{pol}^{stock} and V_{pol}^{stock} designate the PS-g-PEO density, and the concentration and the volume of PS-g-PEO stock solution respectively. V_{Pt}^{stock} targeting $C_{Pt,nps}^{targ}$ can subsequently be computed as

$$V_{Pt}^{targ} \approx \frac{C_{Pt,nps}^{targ}\, (V_{DOS}\, \rho_{pol} + Cm_{pol}^{stock}\, V_{pol}^{stock})}{C_{Pt}^{stock}\, \rho_{pol}} \tag{S18}$$

Assuming again the total volume of co-polymer and DOS used during formulation $V_{pol,DOS}^{tot}$ to be equal to the total volume of the nanoparticles V_{nps}, we eventually derived the experimental PtOEP concentration in the nanoparticles, $C_{Pt,nps}^{exp}$, with Eq.(S20)

$$C_{Pt,nps}^{exp} = \frac{\epsilon_{nps}}{\epsilon_{THF}} \frac{1}{N_a V_{nps}}. \tag{S20}$$

It led us to compute the incorporation yield χ_{nps} of PtOEP from its division by the theoretical concentration, $C_{Pt,nps}^{th}$, which was found to range from 0.33 to 0.82 under the explored conditions.

Figure S7a–d respectively displays the dependence of the experimentally measured diameter, PtOEP incorporation yield, molar absorption coefficient and luminescence quantum yield of the nanoparticles on the experimentally determined PtOEP concentration or the targeted PtOEP concentration. The diameter of the nanoparticles was found to be weakly dependent on the PtOEP concentration; we found 190 nm for the mean value. The PtOEP incorporation yield exhibits a decreasing trend upon increasing the PtOEP concentration. As expected from the Beer-Lambert's law, the molar absorption coefficient linearly depends on the PtOEP concentration in the nanoparticles. Conversely, the luminescence quantum yield of the nanoparticles decreases when the PtOEP concentration within the nanoparticles is increased, as anticipated from an enhanced triplet-triplet desexcitation. Hence, both parameters governing the brightness of the nanoparticles exhibit an opposite dependence on the PtOEP concentration. Therefore, the intermediate concentration of 55 mM was chosen as the optimal targeted PtOEP concentration for the final O_2 nanosensor.

2.1.2 Optimization of the DOS concentration

The influence of the DOS concentration in the nanosensor formulation was then investigated from 3 to 300 μM range. In this experiment, the volume of the PtOEP stock solution was kept constant at 248 μL for targeting 55 mM PtOEP concentration. The different volumes of added DOS are reported in Table S3 together with the measured incorporation yield and brightness. The yield of PtOEP incorporation in the nanoparticles exhibits a maximum at 34 μM DOS concentration.

DOS concentration (μM)	PtOEP incorporation yield (%)	Brightness (mol.L^{-1}.cm^{-1})
3.5	15	1.8×10^4
34	71	1.4×10^5
67	29	2.2×10^5
299	3	4.0×10^3

Table S3: Targeted DOS concentration and volume of DOS stock solution used in the formulation of the nanoparticles; resulting PtOEP incorporation yield and nanosensor brightness.

In fact, we noticed that significative quantities of the platinum complex deposited on the round-bottomed flask at the lowest and highest DOS concentrations, which explains the unusually low incorporation yield for those two conditions. The brightness is here dominated by the molar absorption coefficient. Hence, a weaker incorporation yield, meaning a weaker PtOEP concentration, yields a weaker absorption coefficient and the brightness is maximal at intermediate DOS

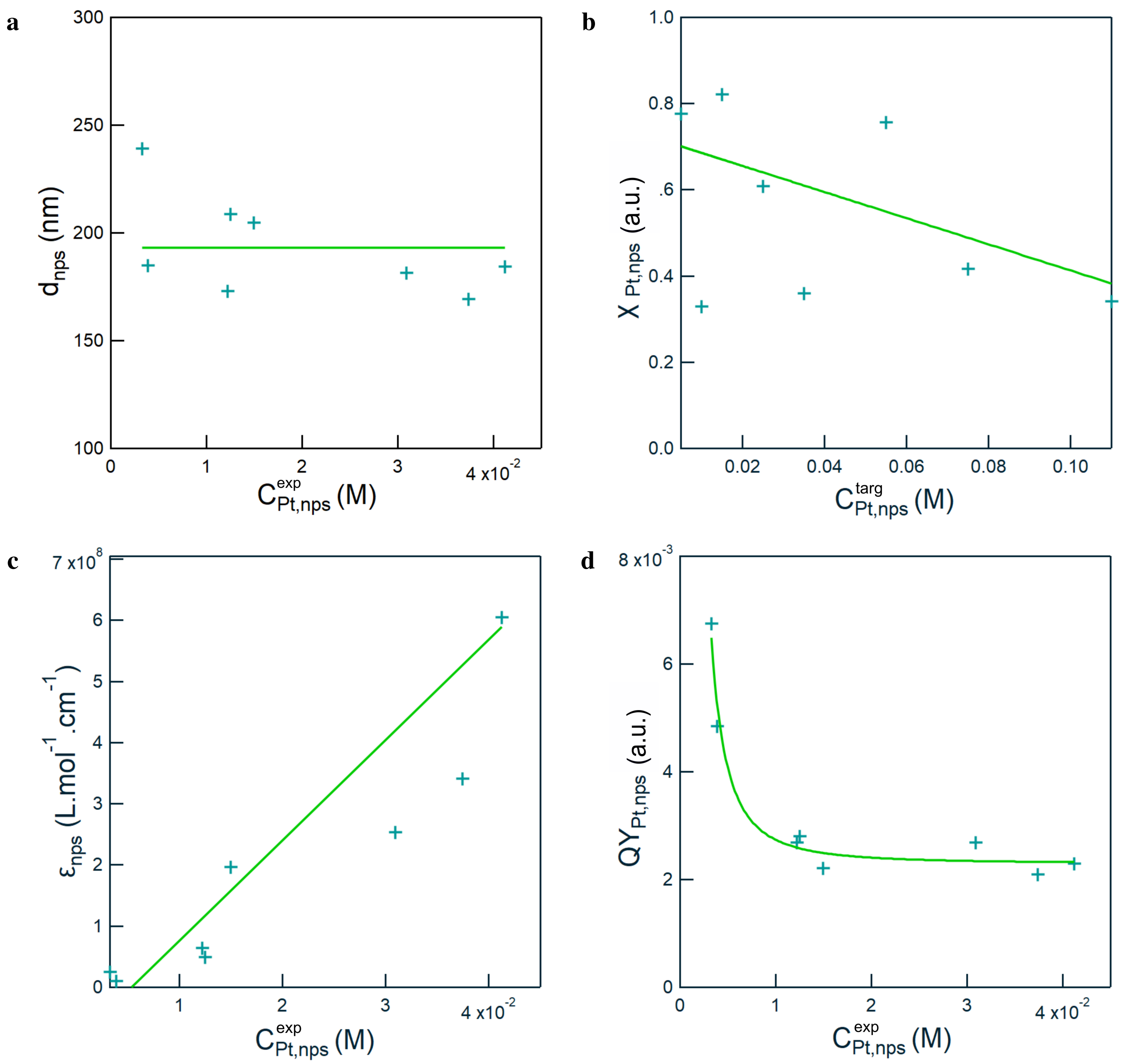

Figure S7: *Optimization of the PtOEP concentration.* **a**: Dependence of the molar absorption coefficient on the experimentally determined PtOEP concentration in the nanoparticles, $C^{exp}_{Pt,nps}$(mol.L^{-1}). Experimental data (cross markers); linear fit ϵ_{nps}(mol^{-1}.L.cm^{-1}) = $1.6\times10^{10} \times C^{exp}_{Pt,nps} - 8.8 \times 10^{7}$ (solid line); **b**: Dependence of the luminescence quantum yield of the PtOEP-labeled nanoparticles on the experimentally determined PtOEP concentration in the nanoparticles, $C^{exp}_{Pt,nps}$(mol.L^{-1}). Experimental data (cross markers); power law fit $\phi(C^{exp}_{Pt,nps})$= 0.002 + $4.16\times10^{-8} \times (C^{exp}_{Pt,nps})^{-2.0}$ (solid line).

concentrations. Therefore, the 34 μM DOS concentration was chosen as the optimal targeted DOS concentration for the nanoparticles.

2.2 Theoretical evaluation of the conformation of the hydrophilic polymer chains in the O_2 nanosensor

The commercial copolymer used to formulate the O_2 nanosensor has known features that enabled us to predict the conformation of its hydrophilic chains around its hydrophobic core from its shape.

It consists in a main chain of polystyrene ($M_{n,PSchain} = 7000$ g/mol) grafted around with 6 chains of polyethylene oxide ($M_{n,PEGchain} = 4500$ g/mol). The total molar mass of the grafted polymer is $M_n = 34000$ g/mol. n_{EG}, the number of ethylene glycol units in one chain of polyethylene glycol, can be derived from Eq.(S21)

$$n_{EG} = \frac{M_{n,PEGchain}}{M_{EGunit}} = \frac{4500}{44} \approx 100 \tag{S21}$$

with M_{EGunit} the molar mass of one ethylene glycol repetitive unit.

We first assess the grafted density $d_{EG,sensor}$, of PEG chains on one nanosensor particle given in Eq.(S22)

$$d_{EG,sensor} = \frac{N_{PEG,tot}}{S_{sensor,tot}} \tag{S22}$$

where $N_{PEG,tot}$ is the total number of polyethylene oxide chains introduced during the synthesis and $S_{sensor,tot}$ is the surface of all the sensors produced.

Designating $N_{pol,tot}$ as the number of grafted polymer molecules in the whole sample, we then derive Eq.(S23)

$$N_{PEG,tot} = 6N_{pol,tot} = 6N_a \frac{m_{pol}}{M_n} \tag{S23}$$

where N_a is the Avogadro constant, and m_{pol} is the total mass of grafted polymer introduced in the synthesis.

Assuming a monodispersion in size for the nanosensors, the surface of all the nanosensors $S_{sensor,tot}$ comes from the number of nanosensors produced N_{sensor} , and the surface of one nanosensor S_{sensor}. N_{sensor} is computed from the total volume of grafted polymer used for nanoprecipitation V_{pol} and the volume of one nanosensor based on the radius of the hydrophobic core, r. V_{pol} can be assessed with the specific mass of the grafted polymer ρ_{pol}, the molar mass of the graft polymer M_n, and the mass of polymer used in the synthesis process, m_{pol}.

$$S_{sensor,tot} = N_{sensor} S_{sensor} = \frac{V_{pol}}{\frac{4}{3}\pi r^3} \times 4\pi r^2 = 3\frac{V_{pol}}{r} = \frac{3m_{pol}}{\rho_{pol} r} \tag{S24}$$

Then, one derives Eq.(S25) for the grafted density $d_{EG,sensor}$, which depends on constants and on the radius of the nanosensor only:

$$d_{EG,sensor} = \frac{N_{PEG,tot}}{S_{sensor,tot}} = 2N_a r \frac{\rho_{pol}}{M_n} \tag{S25}$$

In order to predict the conformation of the hydrophilic chains around the hydrophobic core of the nanosensor, we introduce the dimensionless grafting density σ.[10][11]

$$\sigma = d_{EG,sensor}\, {a_{EGunit}}^2 \tag{S26}$$

where a_{EGunit} is the length of one ethylene glycol unit ($a_{EGunit} \approx 0.3$ nm.[12],[13]). Then, D, the distance between two grafted sites is[10]

$$D = \frac{a}{\sqrt{\sigma}} \tag{S27}$$

$R_f = a\, n_{EG}{}^{\frac{3}{5}}$ designates the Flory's diameter of a polyethylene glycol chain where n_{EG} is the number of ethylene glycol units in one lateral chain of polymer. Here, $R_f = 4.7$ nm. For a sensor with a hydrophobic radius of 70 nm, $D = 1.2$ nm. When $D < R_f$, the polymer coils overlap on the surface. As a consequence, the polymer chains are in a brush regime being stretched over an effective chain length L_{PEG} given in Eq.(S28)

$$L_{PEG} = n_{EG}\, a\, \sigma^{\frac{1}{3}} \tag{S28}$$

We can then compute the dependence of the hydrodynamic diameter $D_{hydro} = 2r + 2L_{PEG}$ on the radius r of the nanosensor core. With $r = 70$ nm measured in CryoTEM experiments and $L_{PEG} = 20$ nm, we retrieved $D_{hydro} = 180$ nm, which is well aligned with the FCS measurements.

2.3 Comparison of the PtOEP photophysical properties in the O_2 nanosensor and in THF

We compared the PtOEP photophysical properties in the O_2 nanosensor and in THF in order to support our protocol to determine the PtOEP incorporation yield used above, and to bring insights for tentatively interpreting the results of the experiments for measuring the luminescence lifetime of the O_2 nanosensor.

2.3.1 Absorption properties

Figure S8a,b displays the wavelength dependence of the molar absorption coefficient of PtOEP in THF and in the O_2 nanosensor in water. The main absorption peaks are found at 380, 509 and 535 nm are similar in both solvents and are in agreement with the literature results.[14] This observation led us to hypothesize that the molar absorption coefficient of the PtOEP complex was similar in THF and in the polystyrene core of the O_2 nanosensor in order to evaluate the PtOEP incorporation yield during nanoprecipitation of the O_2 nanosensor.

2.3.2 Luminescence properties

Figure S9a,b displays the luminescence emission spectra of PtOEP in THF and in the O_2 nanosensor in water upon excitation at 535 nm. We observed an emission band at 644 nm with 35 nm width at half-height, as reported in the literature.[14] The luminescence quantum yield measured in the O_2 nanosensor ($QY_{Pt,nps} = 2.3 \times 10^{-3}$) is slightly higher than the one reported in THF in the litterature ($QY_{Pt,THF} = 1.4 \times 10^{-3}$).[14]

Figure S10a,b eventually displays the luminescence excitation spectra of PtOEP in THF and in the O_2 nanosensor in water upon emission at 644 nm. Since they are similar and comparable with the UV-vis absorption spectra shown in Figure S8a,b, it was concluded that there were no differences of PtOEP morphology of the complex in THF and in the O_2 nanosensor. In particular, no aggregates of PtOEP complexes was concluded to be formed in the O_2 nanosensor.

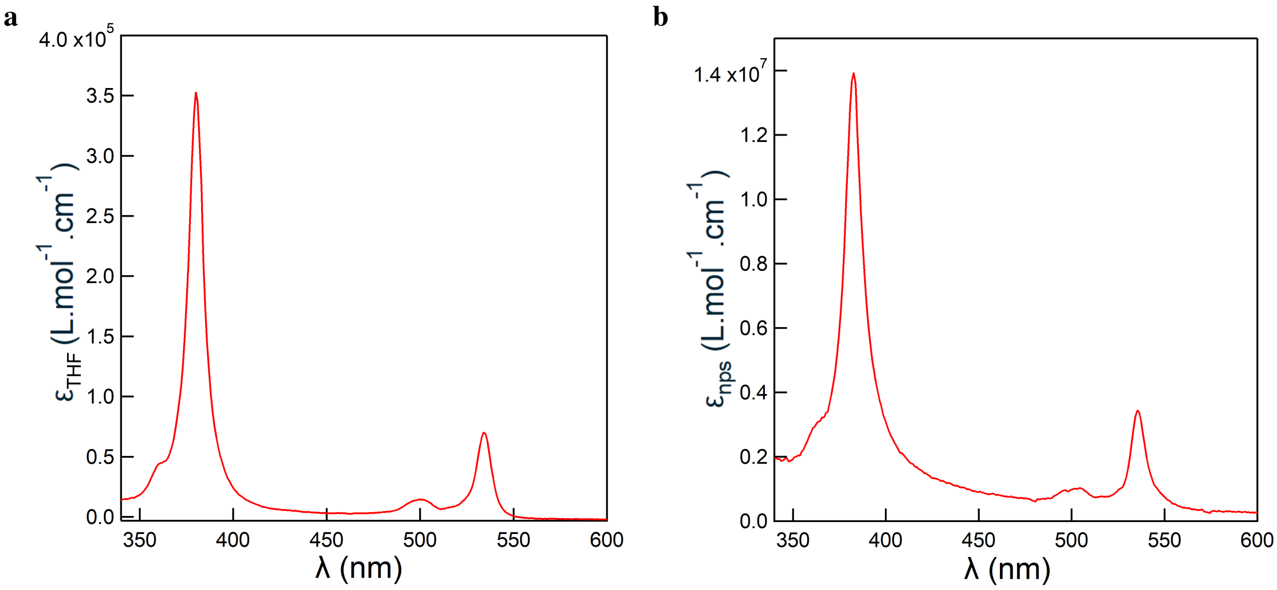

Figure S8: *Absorption properties*. Molar absorption coefficient spectrum $\epsilon_{THF}(\lambda)$ of PtOEP in THF (**a**; $C_{Pt}^{sol} = 1.1\ \mu$M) and in the O_2 nanosensor in water $\epsilon_{nps}(\lambda)$ (**b**; $C_{Pt}^{targ} = 55$ mM, $C_{nps}^{sol} = 1.1 \times 10^{-10}$ M). $T = 293$ K.

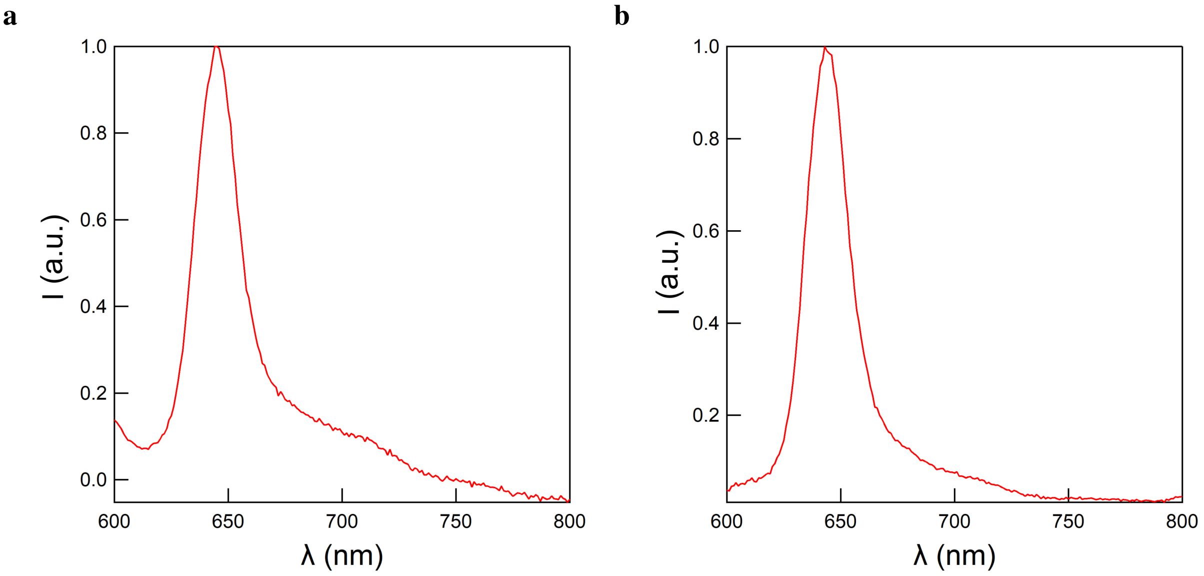

Figure S9: *Luminescence emission properties*. Luminescence emission spectrum $I(\lambda)$ of PtOEP in THF (**a**; $C_{Pt}^{sol} = 1.1\ \mu$M) and in the O_2 nanosensor in water (**b**; $C_{Pt}^{targ} = 55$ mM, $C_{nps}^{sol} = 1.1 \times 10^{-10}$ M). $\lambda_{exc} = 535$ nm, $T = 293$ K.

Figures S11a,b display the measurements of the PtOEP luminescence lifetime performed in THF and in the O_2 nanosensor. Overall, PtOEP first exhibits a longer luminescence lifetime in the nanosensor than in THF. This phenomenon has been accounted for from the difference of the O_2 solubility between water and THF.[15] Yet, one can also notice that at room temperature, the diffusion coefficient of O_2 is much lower in polystyrene (3.1×10^{-11} $m^2.s^{-1}$[16]) than in water (3.1×10^{-9} $m^2.s^{-1}$). Hence, the O_2-induced dynamic quenching of PtOEP luminescence is expected to be weaker and

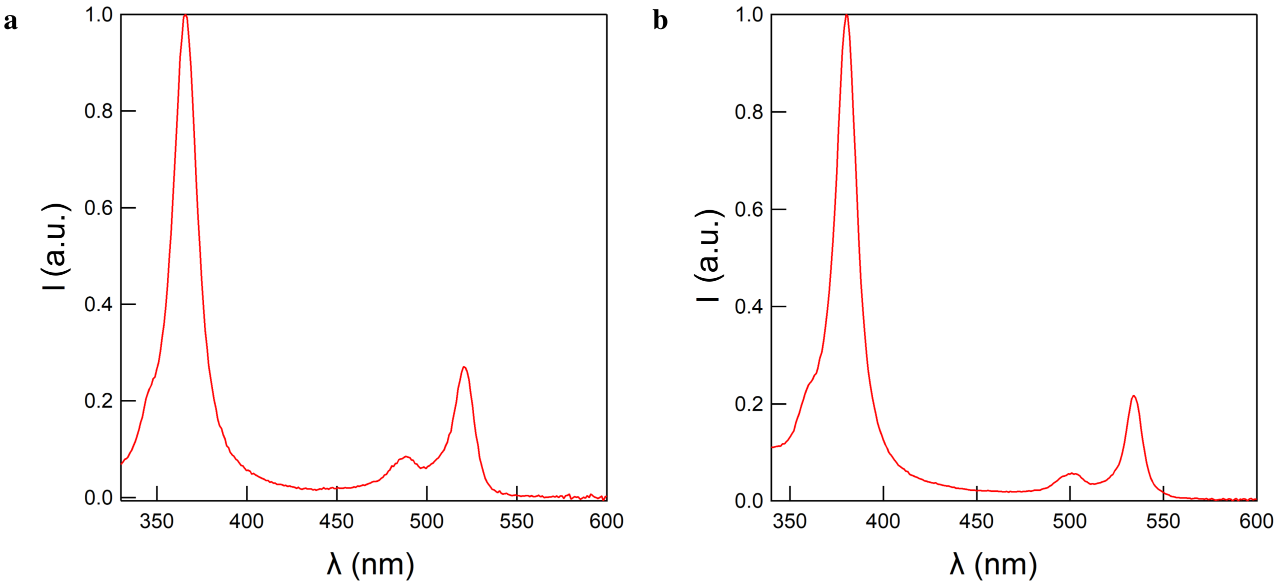

Figure S10: *Luminescence excitation properties.* Normalized luminescence excitation spectrum $I(\lambda)$ of PtOEP in THF (**a**; $C_{Pt}^{sol} = 1.1$ μM) and in the O_2 nanosensor in water (**b**; $C_{Pt}^{targ} = 55$ mM, $C_{nps}^{sol} = 1.1 \times 10^{-10}$ M). $\lambda_{em} = 644$ nm, $T = 293$ K.

the lifetime of its excited state to be enhanced in the nanosensor.

Then, in contrast to the observation made in THF, the frequency-dependence of the luminescence amplitude could not be satisfactorily fitted with Eq.(S5) in the O_2 nanosensor. Such a difference of PtOEP behavior between solvents and polystyrene films was already reported and interpreted as involving triplet-triplet annihilation at the shortest times.[14] In the present case, we also envision that a O_2 gradient dropping from the surface to the core of the O_2 nanosensor could form under the illumination applied during the lifetime measurement. Indeed, when the RIOM protocol was applied with PtOEP-loaded polystyrene microbeads with diameters increasing from 3 to 10 μm,[7] we noticed that the luminescence lifetime rose with increasing microbead diameter (Figure S11c) and from the periphery to the core of 10 μm diameter microbeads (Figure S11d), reaching the 100 μs range expected in anaerobic polystyrene.[14]

To process the data, we phenomenologically applied Eq.(S6) that involves two different luminescence lifetimes and retrieved 1.0×10^{-5} s and 1.5×10^{-6} s associated to 0.11 and 0.67 amplitudes respectively from Figure S11b. These amplitudes were fixed for each fit performed for the characterisation of the sensor. Short lifetime contribute with a higher amplitude to the cutting function. Moreover, RIOM measurements were performed along with lock-in detection on the same sample (See subsection 2.7). Lifetime retrieved by RIOM protocol matched the shorter lifetime measured with lock-in detection. Considering the respective amplitudes of each lifetime from the fitting and the accordance with RIOM protocol, we only used the shorter lifetime for the subsequent calibration and O_2 measurements.

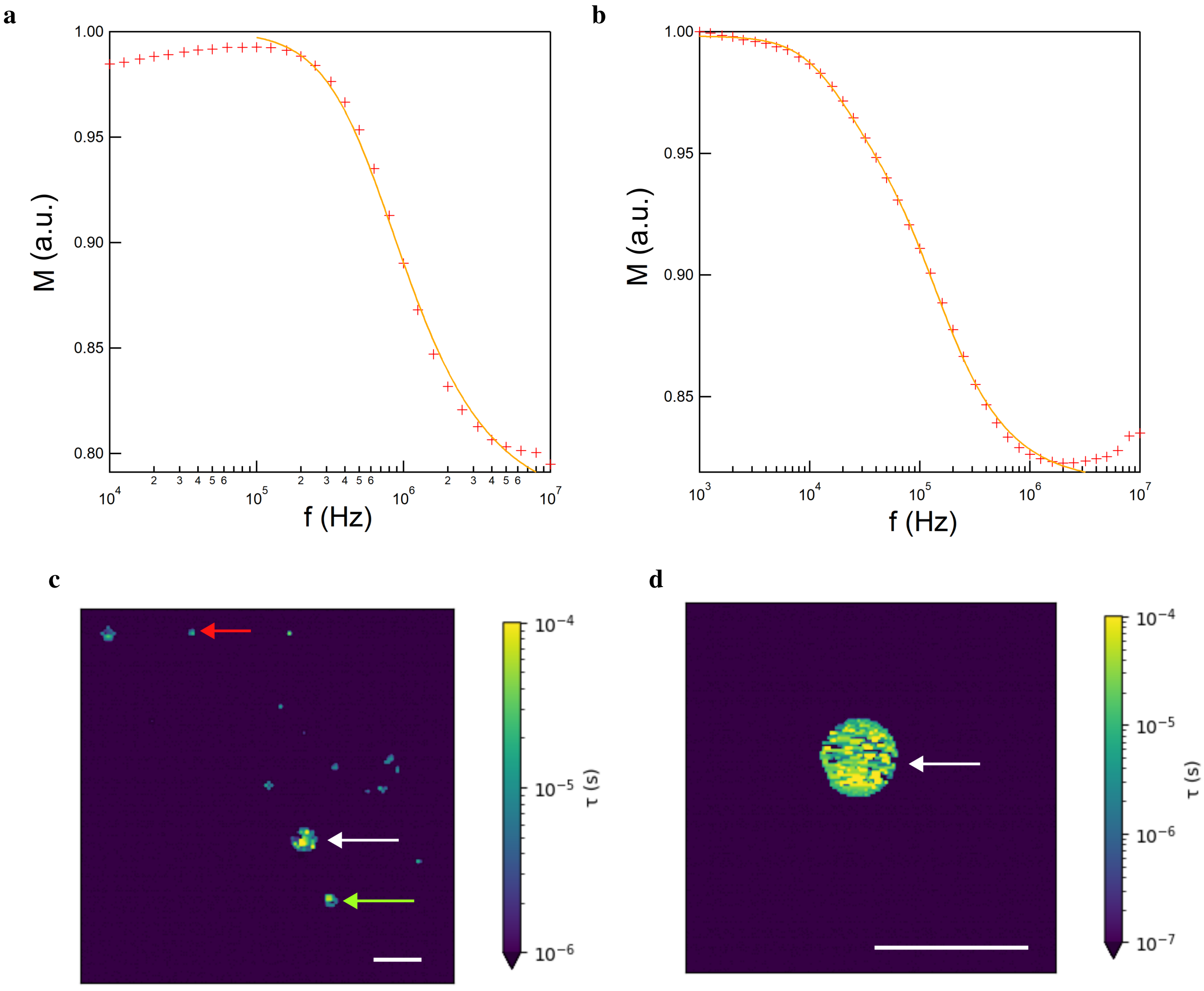

Figure S11: *Luminescence lifetime properties*. Dependence of the normalized amplitude of the modulation M of the PtOEP luminescence in THF (**a**; $C_{Pt}^{sol} = 1.1\ \mu$M) and in the O_2 nanosensor in water (**b**; $C_{Pt}^{targ} = 55$ mM, $C_{nps}^{sol} = 1.1 \times 10^{-10}$ M) on the frequency of the modulated light excitation f. The fits (solid line) of the experimental data (cross markers) with Eqs.(S5) and (S6) yield $a = 0.23$ and $\tau = 0.27\ \mu$s, and $a_1 = 0.11$, $\tau_1 = 1.5\ \mu$s, $a_2 = 0.67$, and $\tau_2 = 10\ \mu$s; **c**,**d**: Representative RIOM maps of the phosphorescence lifetime of 3 (red arrow; **c**), 6 (green arrow; **c**), and 10 (white arrow; **c** and **d**) μm diameter PtOEP-loaded carboxylate polystyrene microbeads. Scale bar: 20 μm. $T = 293$ K.

2.4 Dependence of the luminescence properties of the nanosensor on O_2 concentration

2.4.1 Protocol

The dependence of the brightness and lifetime of the nanosensor on O_2 concentration was assessed by exploiting the quantitative consumption of O_2 by sodium dithionite, which has been reported to occur in a 1:1.8 stoichiometry with

Eqs.(S29,S30).[17]

$$S_2O_4^{2-} + \frac{1}{2}\,O_2 + H_2O \;\rightarrow\; 2\,HSO_3^- \quad \text{(S29)}$$

$$S_2O_4^{2-} + \frac{3}{2}\,O_2 + H_2O \;\rightarrow\; 2\,HSO_4^- \quad \text{(S30)}$$

In practice, different masses of sodium dithionite (around 1 mg) have been individually weighted and stored in independent sealed vials. 1 mL of 20 mM pH = 8 Tris buffer was added to each dithionite vial from which a volume ranging from 10 to 300 μL was immediately pipetted and mixed up with 20 mM pH = 8 Tris Buffer up to 0.96 mL, with 40 μL of nanosensor suspension contained in a 1 mL quartz cuvette. The measurement of the brightness and luminescence lifetime of the nanosensor was then performed at once.

2.4.2 Dependence of the brightness and luminescence lifetime of the nanosensor on O_2 concentration

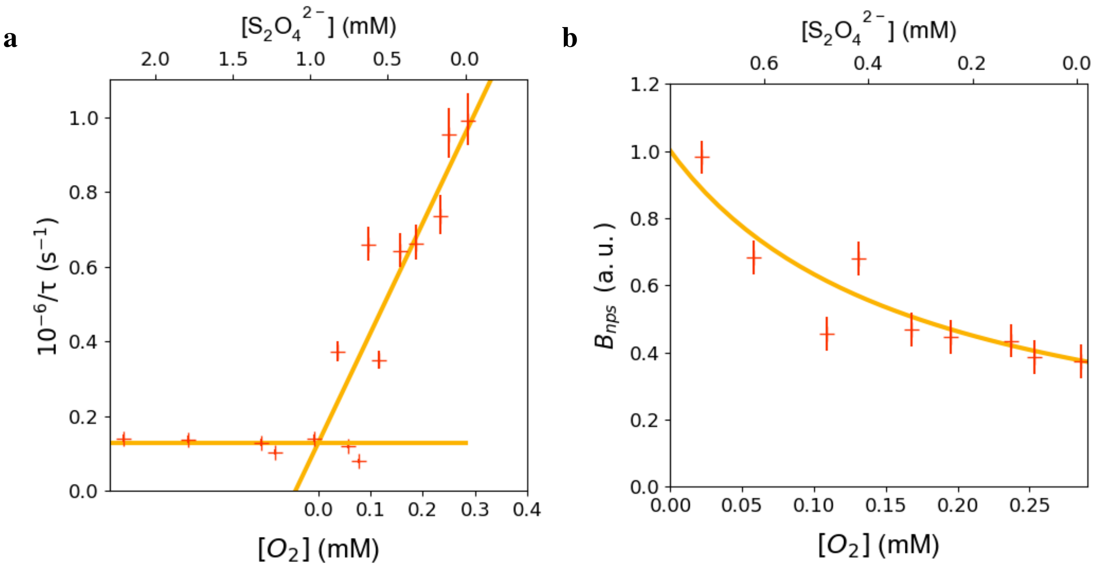

Figure S12: *Calibration of the O_2 nanosensor.* **a**: Dependence of the inverse of the shorter lifetime of the excited state of the PtOEP in the nanosensor on the dithionite concentration in solution and corresponding O_2 concentration. Fits with Eq. (S31) yield the following parameters : $a_1 = 9.75 \times 10^5$ s^{-1}, $b_1 = -1.05 \times 10^9$ s^{-1}.M^{-1}, $a_1' = 1.29 \times 10^5$ s^{-1}, $[S_2O_4^{2-}]_0 = 780$ μM; **b**: Dependence of the normalized amplitude of modulation at 1 kHz of the luminescence response of the platinum complex in the nanosensor on the dithionite concentration in solution and corresponding O_2 concentration. Fit with Eq. (S33) yields the following parameters : $\alpha = 5802$ M^{-1}.

Figure S12a displays the plot of the inverse of the shortest luminescence lifetime of the nanosensor, $1/\tau_1$ denoted as $1/\tau$ for simplification in the following and in the Main Text, against the concentration of added sodium dithionite $[S_2O_4^{2-}]$ and the resulting residual O_2 concentration. Upon increasing the concentration of sodium dithionite, the luminescence lifetime τ first increases and then saturates above a threshold value. Assuming a Stern-Volmer dynamic quenching,[1] this dependence was satisfactorily fitted with a 3-parameter fitting function:

$$\frac{1}{\tau} = a + b[S_2O_4^{2-}],\;\; [S_2O_4^{2-}] < [S_2O_4^{2-}]_0 \;\text{ and }\; \frac{1}{\tau} = a',\;\; [S_2O_4^{2-}] > [S_2O_4^{2-}]_0 \quad \text{(S31)}$$

with $a = 9.75 \times 10^5$ s^{-1}, $b = -1.05 \times 10^9$ s^{-1}.M^{-1}, $a' = 1.29 \times 10^5$ s^{-1}, and $[S_2O_4^{2-}]_0 = 780$ μM.

The intersection of the two lines indicates at which concentration of sodium dithionite the O_2 initially present in the cuvette has been fully consumed. Since the O_2 concentration in water equilibrated with the atmosphere is known, (284 μM at 20 °C[18][19]), it was possible to retrieve 2.9:1 for the stoichiometry of reaction of sodium dithionite with O_2, which differs from the 1.8:1 stoichiometry previously reported.[17] This difference presumably originates from partial decomposition of sodium dithionite in water.[20],[21] Once this stoichiometry was established, we could subsequently retrieve the dependence of the shortest luminescence lifetime of the nanosensor on the O_2 concentration given in Eq. (S32).

$$\frac{1}{\tau}(\mathrm{s}^{-1}) = 2.34.10^9 \times [\mathrm{O_2}](\mathrm{mol}^{-1}.\mathrm{L}) + 1.29.10^5 \tag{S32}$$

Hence, the lifetime τ increases by a factor 10 when shifting from atmospheric conditions to anoxic conditions in the solution.

The same series of experiments enabled us to extract the dependence of the nanosensor brightness on the O_2 concentration, which was considered to mirror the dependence of the amplitude of the luminescence response of the nanosensor at low frequency (1 kHz). Figure S12b displays the O_2 concentration-dependence of the nanosensor brightness normalized by its value at vanishing O_2 concentration, which was satisfactorily fitted with Eq. (S33)

$$B_{nps} = \frac{1}{1 + \alpha[O_2]} \tag{S33}$$

upon yielding $\alpha = 5802$ M^{-1}.

2.5 Impact of the interaction of the O_2 nanosensor with microalgae

The possible impact of the interaction of the O_2 nanosensor with microalgae has been addressed by harnessing optical density measurement for comparing the growth rate of microalgae populations during their exponential growth phase in the presence of carboxylated polystyrene beads either native or loaded with PtOEP. We first proceeded with calibrating the dependence of the optical density (assimilated to absorbance at $\lambda = 685$ nm) on the cell concentration. Hence we suspended in a spectrometer cuvette 100 μL of algae suspension at different cell concentrations determined with an automated cell counter in 2 mL of deionized water (Figure S13a). Then, we measured the optical density of growing cultures every 24 h for 4 days in the presence of the nanoparticle either native without PtOEP or loaded with PtOEP to produce the nanosensor. As displayed in Figure S13b, the experimental data satisfactorily obey an exponential behavior. Moreover, we did not notice any significant different of the growth rate of the microalgae cultivated under both conditions, which has supported the absence of any important toxicity that would be induced by the presence of PtOEP in the medium.

2.6 Measurement of the O_2 flux from a cell population

The evolution of the O_2 concentration has first been studied in the dark after sealing the sample containing microalgae upon considering that its initial value was the one in water equilibrated with the atmosphere: $[O_2]_{\mathrm{atm}} = 284$ μM. After

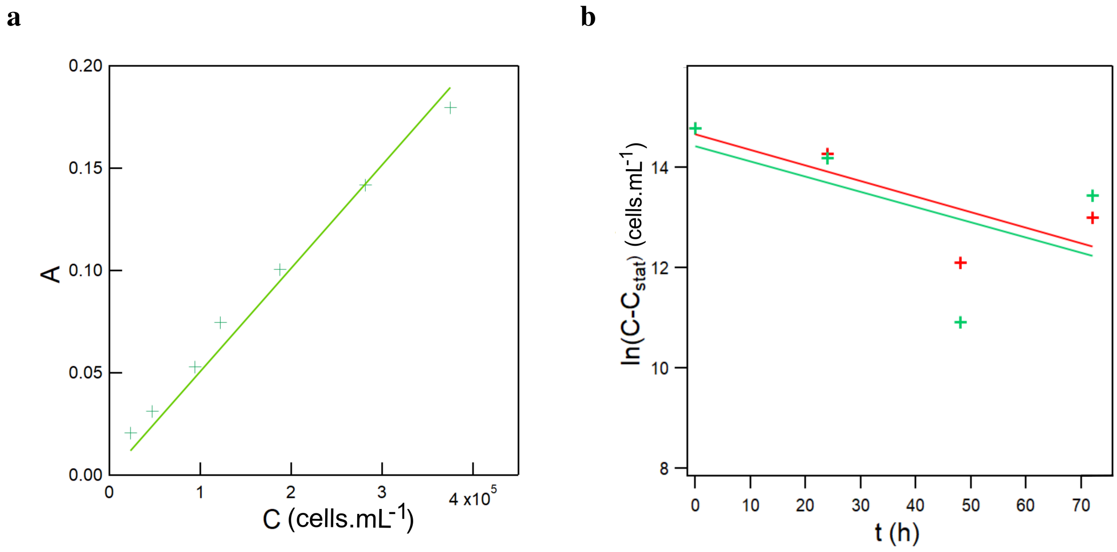

Figure S13: **a**: Dependence of the absorbance of the microalgae suspension at 685 nm on the cell concentration. Experimental data: cross markers, solid line: $A(685) = 5.1 \times 10^{-7} \times C_{algae}$(cells/L) linear fit; **b**: Time evolution of the algae concentration after inoculation of platinum complex-empty (green) or loaded (red) nanoparticles. Experimental data: cross markers, solid line: $ln(C - C_{stat}) = \alpha - \beta t$ linear fit with C_{stat} being the maximum cell concentration achieved during the stationary phase.

$\Delta t = 4$ h in the dark, which promoted respiration and reduced O_2 concentration, we implemented lock-in detection to retrieve $\tau_0 = 5.8 \times 10^{-6}$ s for the luminescence lifetime of the nanosensor, which yielded $[O_2]_0 = 18$ μM from using Eq. (S32). Then, we computed $\Delta[O_2] = [O_2]_{atm} - [O_2]_0$ as the O_2 concentration consumed by the microalgae and extracted a mean dark respiration rate per cell K with Eq. (S34)

$$K = \frac{\Delta[O_2]V_{react}}{C_{cells}V_{culture}\Delta t} = -8.6 \times 10^{-15} \text{ mol.s}^{-1}.\text{cell}^{-1}. \quad \text{(S34)}$$

where V_{react} is the volume of the sealed sample, and $V_{culture}$ is the volume of the cell suspension introduced into the sample at the concentration C_{cells}.

At the end of the latter step, the evolution of the O_2 concentration has then been studied under illumination of the sealed sample containing the microalgae with a white broad light (maximum emission is 685 nm; see Figure S2) at 50 μmol photons.m^{-2}.s^{-1} intensity. The luminescence lifetime of the sample and the associated O_2 concentration were assessed every $\Delta t' = 30$ min over 2 h. The O_2 concentration $[O_2]_i$ was shown to increase on time and then to saturate (Figure S14a). Conversely, the mean O_2 flux K_i retrieved from $\Delta[O_2]_i = [O_2]_{i+1} - [O_2]_i$ between two time points with Eq. (S35) first grew and then dropped as shown in Figure S14b.

$$K_i = \frac{\Delta[O_2]_i V_{react}}{C_{cells}V_{culture}\Delta t'} \quad \text{(S35)}$$

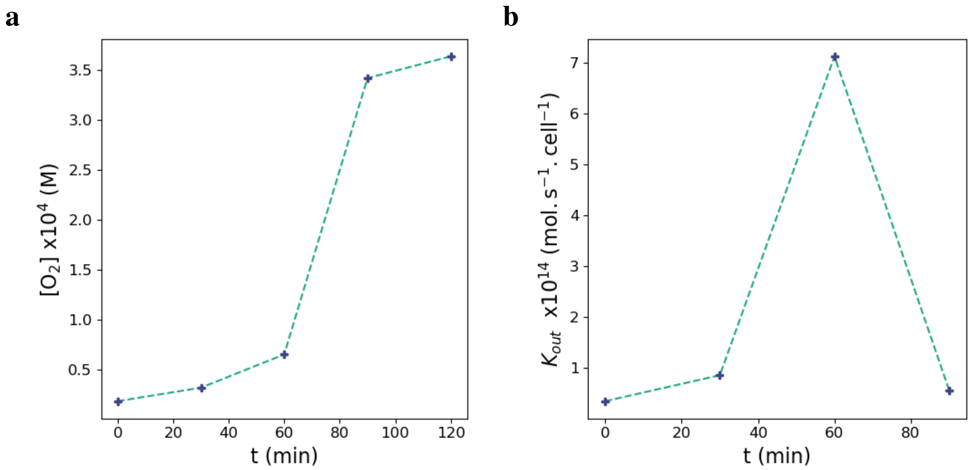

Figure S14: *O_2 Production from a batch of microalgae cells in a sealed sample.* **a**: Time evolution of the O_2 concentration during illumination with a 685 nm light at 50 μmol photons.m^{-2}.s^{-1} intensity; **b**: Corresponding time evolution of the mean O_2 production flux for one cell of *Chlamydomonas reinhardtii* as retrieved from Eq. (S35).

2.7 Determination of the confidence interval of the lifetime measurement with the RIOM protocol

In order to establish the confidence interval of the lifetime measurements performed with RIOM, we imaged an agarose pad with embedded O_2 nanosensor produced as described in the Main Text with the same RIOM protocol used for single cell experiments reported in paragraph 1.2.3.3.

The imaged sample was produced upon adding 100 μL of freshly prepared 1 mM sodium dithionite in 20 mM pH = 8 Tris buffer, before sealing the sample in order to consume O_2 similarly to dark respiration of microalgae. The RIOM acquisition protocol was then applied on the sample. We first fitted the dependence of the RIOM signal over the whole field of view on the frequency of modulated light. Hence, we extracted the luminescence lifetime of the nanosensor τ = (4.8 $\pm$ 0.5)$\times$ 10^{-6} s (Figure S15a), which was found in line with the luminescence lifetime of the nanosensor assessed on the whole field of view with lock-in detection τ = (5.6$\pm$0.7)$\times$ 10^{-6} s (Figure S15b).

We then divided the field of view of the RIOM image in 16 frames (binning 128) and individually retrieved the luminescence lifetime of the nanosensor in each frame in order to extract the standard deviation of the lifetimes over all the frame (Figure S15c). The mean lifetime and the standard deviation retrieved with RIOM over the field of view were $\tau = 5.1 \times 10^{-6}$ s and $\sigma = 0.7 \times 10^{-6}$ s respectively. The confidence interval was then established as $2\sigma = 1.4 \times 10^{-6}$ s (confidence level of 68%).

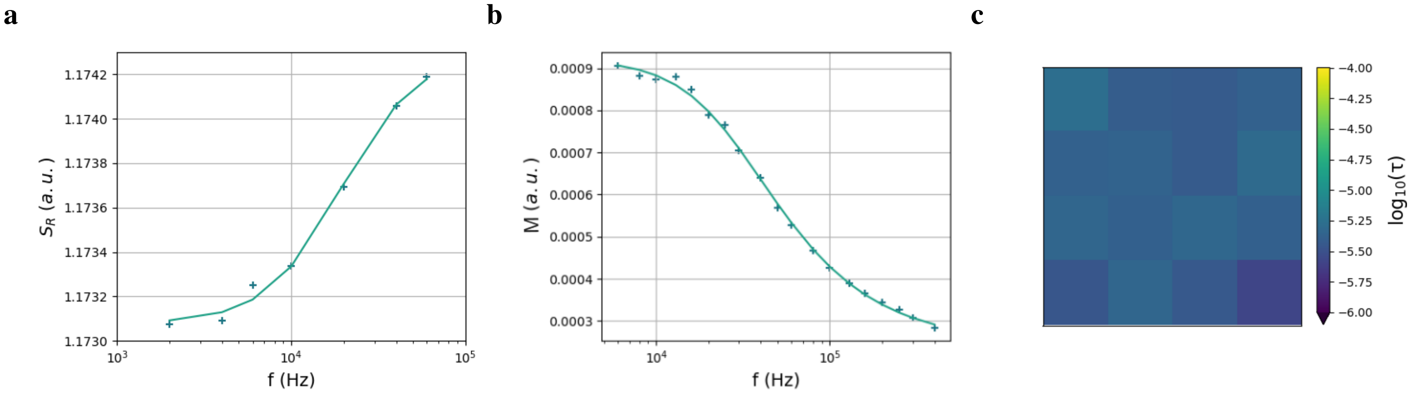

Figure S15: *Measurement of the nanosensor luminescence lifetime and its confidence interval with the RIOM protocol.* **a**,**b**: Measurement of the luminescence lifetime of the O_2 nanosensor embedded in an agarose pad without microalgae either by using the RIOM protocol upon analyzing the whole field of view (**a**) or Lock-in detection (**b**). Cross markers: experimental points; lines: fits with Eq.(S11) (**a**) and Eq.(S5) (**b**). Fitting parameters can be found in Table S1; **c**: Map of luminescence lifetimes retrieved with the RIOM protocol over the 16 segmented frames.

2.8 Measurement of the function of the photon collection of the epifluorescence microscope

The photon collection of the epifluorescence microscope depends on the depth z from the focal plan along the optical axis. For evaluating the associated function, we deposited a monolayer of 3 μm PtOEP-loaded polystyrene microbeads on a 250 μm-thick agarose pad. This sample was imaged and the mean luminescence over a square area centered on a luminescent bead was recorded as a function of the depth z from the focal plan (Figure S16).

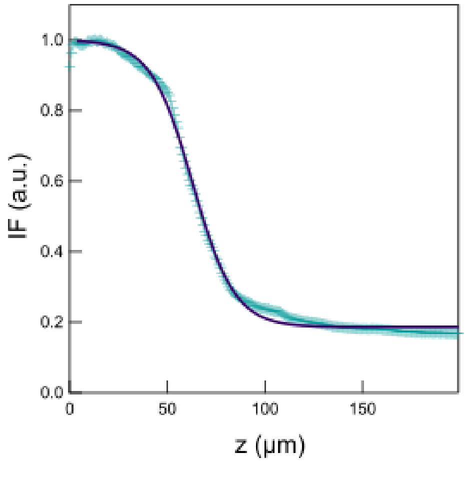

Figure S16: *Depth of focus z-dependence of the normalized luminescence emitted by a 3 μm PtOEP-loaded polystyrene bead.* Cross markers: experimental points; solid line: fit with Eq. (S36).

The photon collection function of the epifluorescence microscope was eventually obtained from satisfactorily fitting the depth of focus z-dependence of the normalized luminescence with the sigmoidal function given in Eq.(S36)

$$IF(z) = 1 - \frac{B}{1 + e^{\frac{z_0 - z}{A}}} \tag{S36}$$

with A = 10.7 μm, B = 0.8 (a.u.) and z_0 = 63 μm.

3 Theoretical computation of 3D profiles of the O_2 concentration around a single cell

3.1 Modeling

In our series of single cell experiments, the observed cell was deposited on a 200 μm-thick agarose pad containing the O_2 nanosensor and the sample was sandwiched between two glass slides. To proceed with the geometrical description for modeling, we considered that the cell (i) was facing an impermeable surface (the coverslip); (ii) could adopt various geometries (hemispherical, cylindrical and spherical) at constant volume.[22],[23] We further considered the diffusion coefficient of O_2 in agar to be identical in agarose and in water[24],[25] so as to assume O_2 diffusion to occur in a homogeneous hemispace.

3.2 Extraction of an analytic law for O_2 diffusion upon assuming axial symmetry of the O_2 concentration profile

We first considered that the cell uniformly emitted O_2 from its surface exposed to the agarose pad.

3.2.1 Numerical simulations

Numerical simulations of concentrations profiles were performed using COMSOL Multiphysics 6.3 software. Here, a two dimension space was used to solve the mass transport equation with two dimension axial symmetry (Figure S17a). Hemispherical, cylindrical and spherical geometries were introduced to model the cell located in the (r, z) plan at the coordinate (0,0) (for hemisphere and cylinder) or at the coordinate (0,r_0) (for sphere of radius r_0). The mesh was designed with appropriate resolution to account for flux emission from the cell surface and geometrical restrictions at certain locations in the two dimension space (Figure S17b).

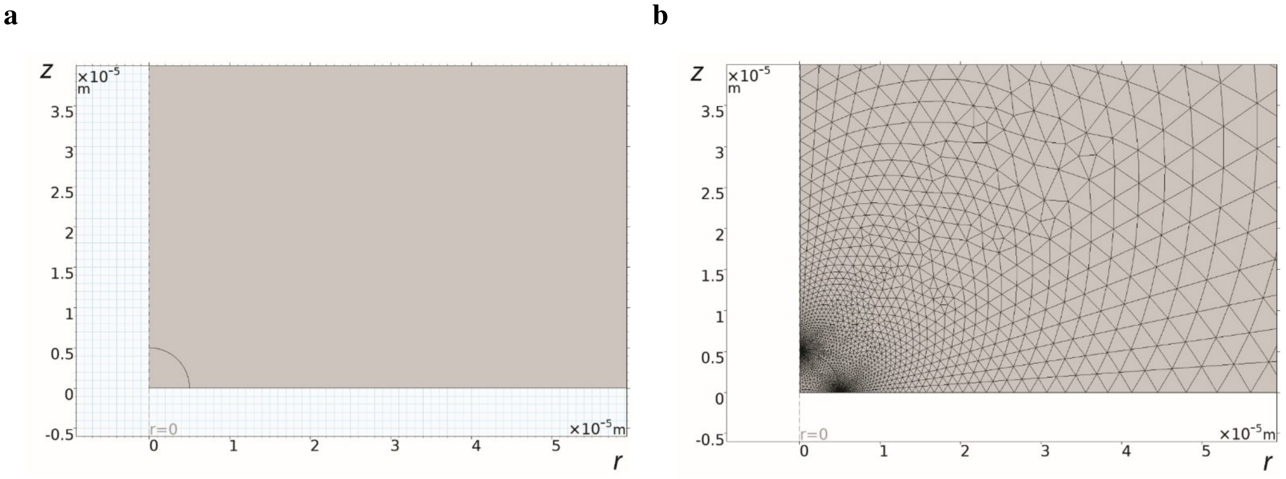

Figure S17: *Two dimension space used for numerical simulations.* **a**: Geometry in (r, z) plan. The alga is assimilated to a 5 μm-radius hemisphere; **b**: Corresponding mesh used for numerical simulations.

C and D designating the concentration and the diffusion coefficient of O_2 respectively, the mass transport equation was solved by finite elements using the diluted species component of COMSOL and considering the following:

1. The diffusion equation : $\frac{\partial C}{\partial t} = -D\nabla^2 C$ in all the space;

2. The initial condition : $t = 0$, $C = C_0$ in all the space;

3. The boundary conditions. Below, n indicates we consider the flux normal to the cell surface :

   (a) $-n\, D\nabla C = J$ on the cell surface with a S area and $J = \frac{K}{S}$ with K the O_2 flux;

   (b) $-n\, D\nabla C = 0$ at the exterior boundaries of space.

The O_2 spatial distribution in steady state was simulated for three shapes of diffusing objects : an hemisphere, a cylinder and a sphere (Figure S18a,b,c). The flux out of the object was kept constant for all geometries with $K = k\,V$ with $k = 26$ mol.m^{-3}.s^{-1} the volumetric flux, and $V = 2.6.10^{-16}$ m^3, the volume of the object. The O_2 concentration profiles along r for different z were extracted (Figure S18 d,e,f).

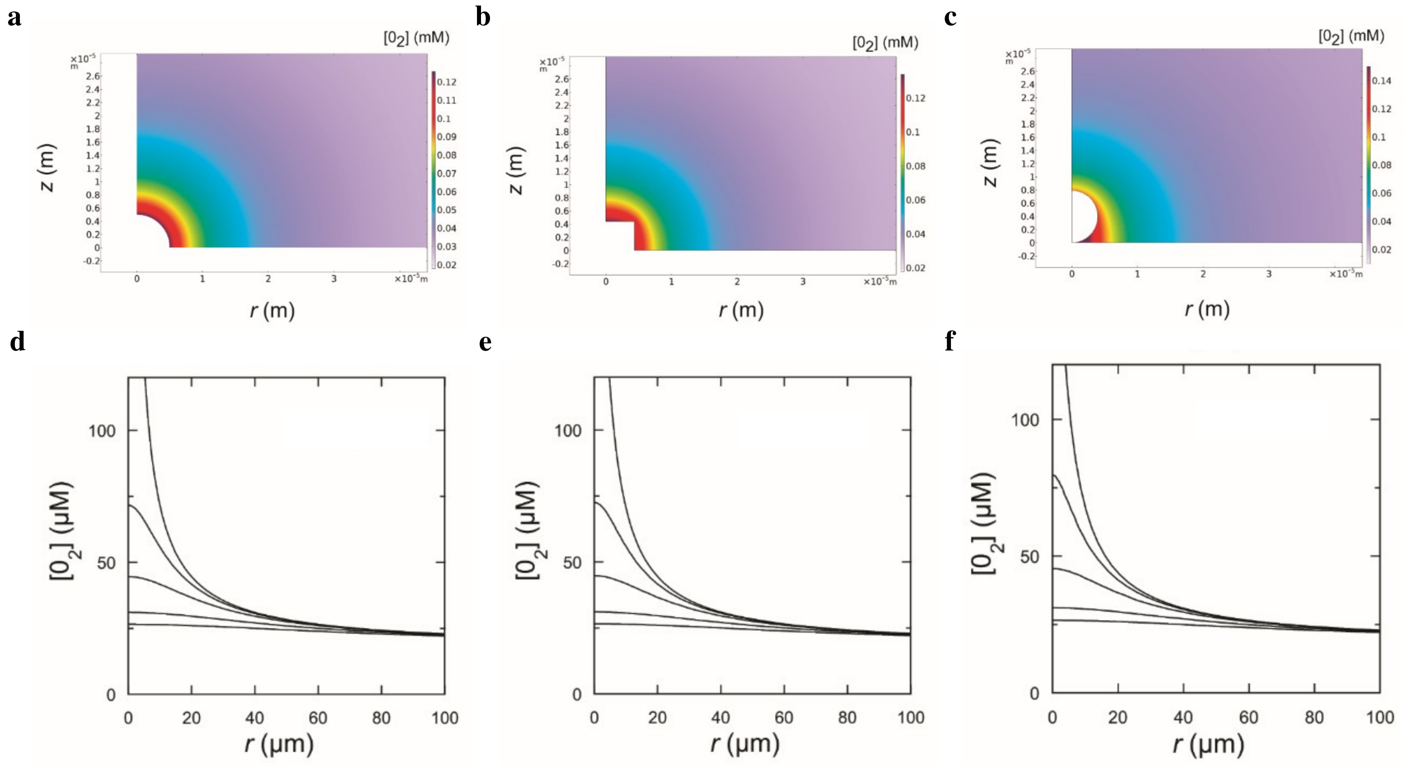

Figure S18: *Comparision of the O_2 concentration spatial distribution in steady-state for different geometries of the diffusing object.* **a**–**c**: Steady-state O_2 concentration two dimensions maps in (r,z) plan simulated for hemispherical (**a**), cylindrical (**b**), and spherical (**c**) geometries; **d**–**f**: Corresponding profiles established at $z = 0$, $10,\ 20,\ 40$ and $60\ \mu$m for hemispherical (**d**), cylindrical (**e**), and spherical (**f**) geometries. $V = 2.6.10^{-16}$ m^3, $k = 26$ mol.m^{-3}.s^{-1}, $C_0 = 18.10^{-6}$ M.

The profiles for each geometry at different z can be compared. At the distance from the cell where the RIOM

measurement has been performed to extract the cellular O_2 flux, there is no significant dependence of the O_2 concentration profile on the geometry of the emitting object (Figure S19).

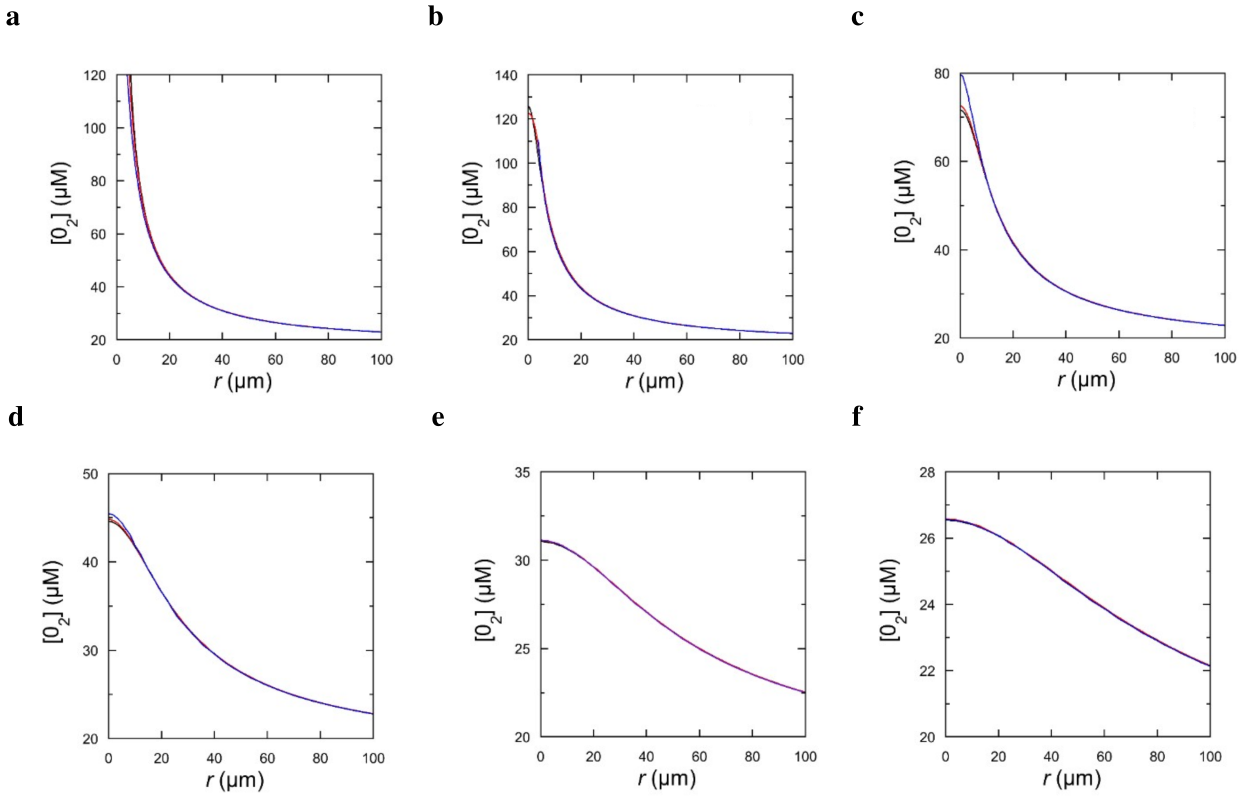

Figure S19: *Steady-state O_2 concentration profiles simulated for different geometries.* Profile along r at $z = 0$ μm (**a**), $z = 5$ μm (**b**), $z = 10$ μm (**c**), $z = 20$ μm (**d**), $z = 40$ μm (**e**), $z = 60$ μm (**f**). Emitting object is hemispherical (black), cylindrical (red) and spherical (blue). $V = 2.6 \times 10^{-16}$ m^3, $k = 26$ mol.m^{-3}.s^{-1}, $C_0 = 18 \times 10^{-6}$ M.

Finally, the dynamics for establishing and disrupting the O_2 spatial distribution upon turning on and off illumination was investigated. The evolution of the O_2 concentration profile along r was assessed at $t = 0.1, 1, 10, 100$ and 200 s. Our simulations displayed in Figure S20 show that the O_2 concentration profile is fully established within 1 s and disappears within the same time.

3.2.2 An analytic fitting function for the O_2 concentration profile

The O_2 cellular flux has been retrieved from fitting the r-dependence of the O_2 concentration profile in the cell plan.

The expression of the analytic fitting function given in Eq. (S37) for the O_2 concentration profile around a single cell has already been established.[26]

$$[O_2](r) = \frac{K}{4\pi D_{O_2}\sqrt{r^2 + z^2}} + C_0 \tag{S37}$$

In contrast, no such analytic fitting function is currently available for an hemispheric geometry with the present border conditions. Considering the mathematical structure of Eq. (S37) , we suggested the analytic function to adopt the

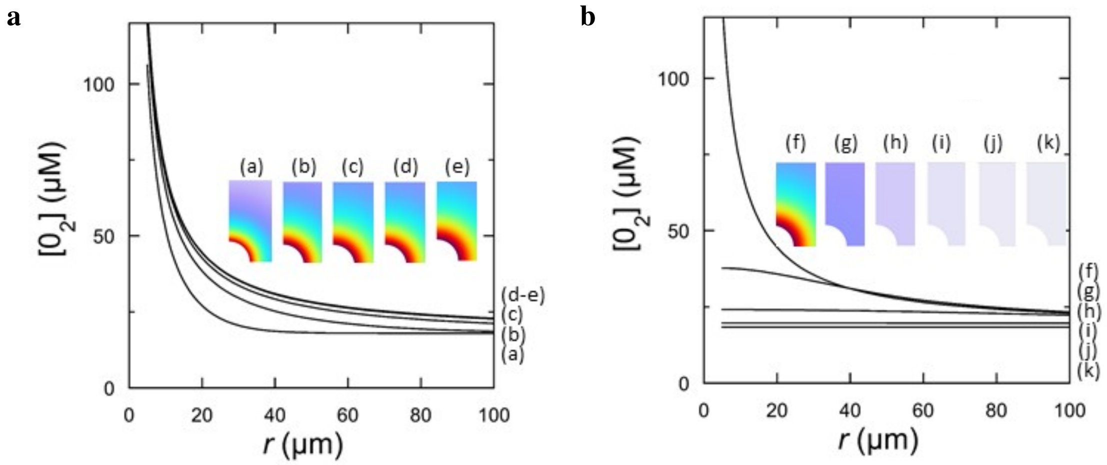

Figure S20: *Dynamics of the O_2 concentration profiles.* Concentration profiles simulated for an hemisphere of 5 μm radius and in inserts, corresponding 2D maps in (r,z) plan. **a**: Concentration profiles after initiating the flux at t = 0.1 (a), 1 (b), 10 (c), 100 (d) and 200 (e) s; **b**: Concentration profiles after interruption of the flux previously established in the steady state: t = 0 (f), 0.1 (g), 1 (h), 10 (i), 100 (j) and 200 (k) s. $V = 2.6 \times 10^{-16}$ m^3, $k = 26$ mol.m^{-3}.s^{-1}, $C_0 = 18 \times 10^{-6}$ M.

following expression :

$$[O_2](r) = \frac{\alpha\, K}{2\pi D_{O_2}\sqrt{r^2+z^2}} + C_0 \qquad \text{(S38)}$$

Simulated data at different fixed z for an hemisphere with a radius of 5 μm were then fitted in order to extract the α parameter (Figure S21), which resulted in eventually yielding the final analytic expression :

$$[O_2](r) = \frac{0.94\, K}{2\pi D_{O_2}\sqrt{r^2+z^2}} + C_0 \qquad \text{(S39)}$$

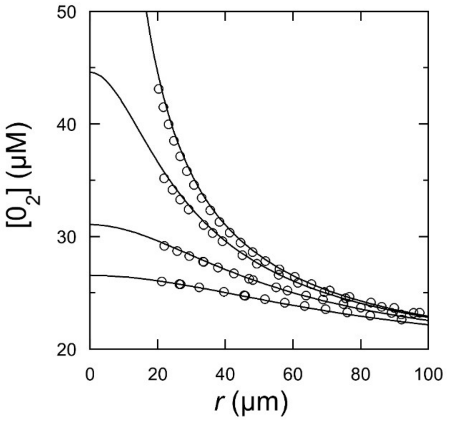

Figure S21: *Extraction of an analytic fitting function for the O_2 concentration profile.* Comparison between steady-state simulated concentration profiles (lines) and Eq. (S39) (open circles) for an hemisphere of 5 μm radius: z = 0, 20, 40, and 60 μm (top to bottom). $V = 2.6 \times 10^{-16}$ m^3, $k = 26$ mol.m^{-3}.s^{-1}, $C_0 = 18 \times 10^{-6}$ M.

3.3 Extraction of the O_2 concentration profile upon leveling off the assumption of axial symmetry

In this subsection, we assumed that the centers of cellular production and consumption of O_2 were not anymore co-localized as it could result from an asymmetric distribution of the chloroplast and the mitochondria within the cell.

3.3.1 Numerical simulations

Numerical simulations of concentrations profiles were performed using COMSOL Multiphysics 6.3 software. Here, a 3D space (x,y,z) with hemispherical geometry was used. The cell was assimilated to an hemisphere with a radius of 5 μm at the coordinates (0,0,0) (Figure S22 a). To model the anisotropy of cellular production and consumption of O_2 within the cell, two active but geometrically opposite surfaces (from quarters) were defined on the hemisphere to consume and produce O_2 respectively (Figure S22 b). One species was considered for modeling O_2 diffusion at C concentration with D diffusion coefficient. The mass transport equation was solved by finite elements using the diluted species component of COMSOL and considering all the conditions presented in section 3.2 and the following :

1. The cell boundary conditions:

   (a) $C = 0$ on the cell surface quarter that consumes O_2;

   (b) $J_{in} = J_{out}$ equality between incoming and outcoming O_2 fluxes at the two opposite quarters of the simulated cell.

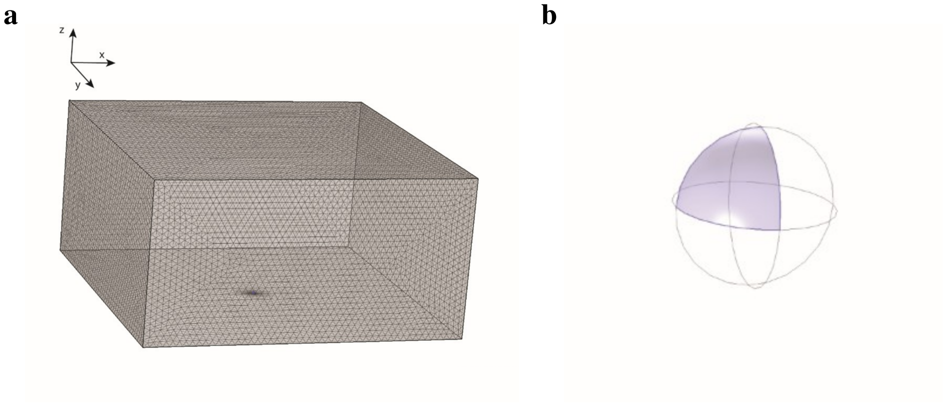

Figure S22: *Parameters for 3D numerical simulations.* **a**: Example of the 3D space used for hemispherical geometry. Hemisphere (blue) of 5 μm radius in (x,y,z) space of (1000 x 1000 x 500 μm) dimensions. Corresponding mesh used for numerical simulation; **b**: Definition in 3D space of a quarter of the simulated cell.

3.3.2 Results

Figure S23 evidences the anisotropy in the profile of extracellular O_2 concentration resulting from antagonist fluxes at the chloroplast and mitochondria, located on opposite quarters of the cells. The simulation shows that the O_2 consumption flux at the surface of the cell is diffusion-limited. Considering the conditions applied to the surface of the cell and the

concentration around the cell, the maximum surfacic consumption flux is 7.2×10^{-6} mol.s^{-1}.m^{-2}, corresponding to $K = -1.1 \times 10^{-15}$ mol.s^{-1}.cell^{-1} by assimilating the cell to a 5 μm radius hemisphere. This diffusion-limited flux corresponds to the order of magnitude of the fluxes retrieved with DCMU conditioning.

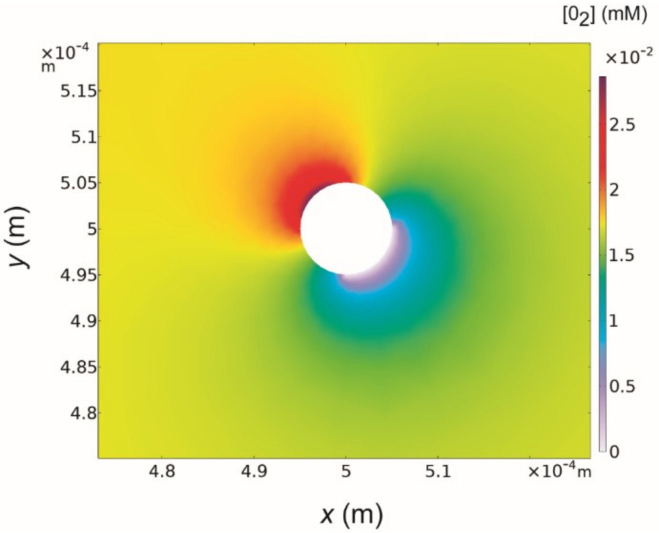

Figure S23: *Steady-state O_2 concentration profiles simulated for hemispherical geometry in 3D space.* 2D map at $z = 0$ in (x,y) plan illustrating the maximal consuming flux at one quarter and the corresponding emitting flux at the opposite quarter. $V = 2.6 \times 10^{-16}$ m^3, $r_0 = 5$ μm, $K_{in} = K_{out} = 1$ mol.m^{-3}.s^{-1}, $C_0 = 18 \times 10^{-6}$ M.

References

[1] Bernard Valeur and M. N. Berberan-Santos. *Molecular fluorescence: principles and applications*. Wiley-VCH Verlag GmbH & Co. KGaA, Weinheim, Germany, second edition edition, 2013.

[2] Samuel T. Hess, Shaohui Huang, Ahmed A. Heikal, and Watt W. Webb. Biological and Chemical Applications of Fluorescence Correlation Spectroscopy: A Review. *Biochemistry*, 41(3):697–705, January 2002.

[3] A. Estévez-Torres, C. Gosse, T. Le Saux, J.-F. Allemand, V. Croquette, H. Berthoumieux, A. Lemarchand, and L. Jullien. Fourier analysis to measure diffusion coefficients and resolve mixtures on a continuous electrophoresis chip. *Analytical Chemistry*, 79(21):8222–8231, November 2007.

[4] R.F. Kubin and A.N. Fletcher. Fluorescence quantum yields of some rhodamine dyes. *Journal of Luminescence*, 27(4):455–462, December 1982.

[5] Albert M. Brouwer. Standards for photoluminescence quantum yield measurements in solution (IUPAC Technical Report). *Pure and Applied Chemistry*, 83(12):2213–2228, August 2011.

[6] Aliénor Lahlou, Hessam Sepasi Tehrani, Ian Coghill, Yuriy Shpinov, Mrinal Mandal, Marie-Aude Plamont, Isabelle Aujard, Yuxi Niu, Ladislav Nedbal, Dusan Lazár, Pierre Mahou, Willy Supatto, Emmanuel Beaurepaire, Isabelle Eisenmann, Nicolas Desprat, Vincent Croquette, Raphaël Jeanneret, Thomas Le Saux, and Ludovic Jullien. Fluorescence to measure light intensity. *Nature Methods*, 20(12):1930–1938, December 2023.

[7] Hélène Merceron, Ian Coghill, Aliénor Lahlou, Marie-Aude Plamont, Ludovic Jullien, and Thomas Le Saux. Periodic Light Modulations for Low-Cost Wide-Field Imaging of Luminescence Kinetics Under Ambient Light. *Advanced Science*, 12(10):2413291, March 2025.

[8] S.W. Jeffrey and G.F. Humphrey. New spectrophotometric equations for determining chlorophylls a, b, c1 and c2 in higher plants, algae and natural phytoplankton. *Biochemie und Physiologie der Pflanzen*, 167(2):191–194, 1975.

[9] Giuseppe Zucchelli, Robert C. Jennings, Flavio M. Garlaschi, Gianfelice Cinque, Roberto Bassi, and Oliviero Cremonesi. The Calculated In Vitro and In Vivo Chlorophyll a Absorption Bandshape. *Biophysical Journal*, 82(1):378–390, January 2002.

[10] P. G. De Gennes. Conformations of Polymers Attached to an Interface. *Macromolecules*, 13(5):1069–1075, September 1980.

[11] Marceau Hénot, Alexis Chennevière, Eric Drockenmuller, Kenneth Shull, Liliane Léger, and Frédéric Restagno. Influence of grafting on the glass transition temperature of PS thin films. *The European Physical Journal E*, 40(1):11, January 2017.

[12] F Oesterhelt, M Rief, and H E Gaub. Single molecule force spectroscopy by AFM indicates helical structure of poly(ethylene-glycol) in water. *New Journal of Physics*, 1:6–6, January 1999.

[13] Devika B Chithrani. Polyethylene Glycol Density and Length Affects Nanoparticle Uptake by Cancer Cells. *Journal of Nanomedicine Research*, 1(1), October 2014.

[14] A.K. Bansal, W. Holzer, A. Penzkofer, and Taiju Tsuboi. Absorption and emission spectroscopic characterization of platinum-octaethyl-porphyrin (PtOEP). *Chemical Physics*, 330(1-2):118–129, November 2006.

[15] Michela Quaranta, Michael Murkovic, and Ingo Klimant. A new method to measure oxygen solubility in organic solvents through optical oxygen sensing. *Analyst*, 138(21):6243–6245, 2013. Publisher: The Royal Society of Chemistry.

[16] Maria Nowakowska, Jan Najbar, and Bolesław Waligóra. Fluorescence quenching of polystyrene by oxygen. *European Polymer Journal*, 12(6):387–391, 1976.

[17] T. Zhimin, J. Goodisman, and A.K. Souid. Oxygen measurement via phosphorescence: Reaction of sodium dithionite with dissolved oxygen. *Journal of Physical Chemistry A*, 112:1511–1518, 2008.

[18] Hiroshi Miyamoto, Yuri Yampolski, and Colin L. Young. IUPAC-NIST Solubility Data Series. 103. Oxygen and Ozone in Water, Aqueous Solutions, and Organic Liquids (Supplement to Solubility Data Series Volume 7). *Journal of Physical and Chemical Reference Data*, 43(3):033102, September 2014.

[19] Bruce B. Benson and Daniel Krause. The concentration and isotopic fractionation of gases dissolved in freshwater in equilibrium with the atmosphere. 1. Oxygen. *Limnology and Oceanography*, 25(4):662–671, July 1980.

[20] A. F Holleman, Egon Wiberg, and Nils Wiberg. *Lehrbuch der anorganischen Chemie*. De Gruyter, Berlin; New York, 1985.

[21] OECD. SIDS Dossier on Sodium Disulphite. Technical report, April 2006.

[22] Ian Streeter and Richard G. Compton. Diffusion-Limited Currents to Nanoparticles of Various Shapes Supported on an Electrode; Spheres, Hemispheres, and Distorted Spheres and Hemispheres. *The Journal of Physical Chemistry C*, 111(49):18049–18054, December 2007.

[23] John Crank. *The mathematics of diffusion*. Clarendon Press, Oxford, 2. ed., reprint edition, 1976.

[24] Niels Peter Revsbech. Diffusion characteristics of microbial communities determined by use of oxygen microsensors. *Journal of Microbiological Methods*, 9(2):111–122, February 1989.

[25] Paul Van Der Meeren, Dries De Vleeschauwer, and Pierre Debergh. [Determination of oxygen profiles in agar-based gelled in vitro plant tissue culture media. *Plant Cell, Tissue and Organ Culture*, 65(3):239–245, 2001.

[26] Shigeharu Kihara, Daniel A. Hartzler, and Sergei Savikhin. Oxygen Concentration Inside a Functioning Photosynthetic Cell. *Biophysical Journal*, 106(9):1882–1889, May 2014.
